# Supplementary material for: A soft thermal sensor for the continuous assessment of flow in vascular access
Source: Nat Commun. 2025 Jan 2;16:38. doi: 10.1038/s41467-024-54942-3 (PMC11696732; doi:10.1038/s41467-024-54942-3)
Supplement: Supplementary file 1 — Supplementary Information [file 41467_2024_54942_MOESM1_ESM.pdf]

Supplementary Figures

A soft thermal sensor for the continuous assessment of flow in vascular access

Yujun Deng, Hany Arafa, Tianyu Yang, Hassan Albadawi, Richard J. Fowl, Zefu Zhang, Viswajit Kandula, Ashvita Ramesh, Chase Correia, Yonggang Huang, Rahmi Oklu,* John A. Rogers,* and Andrea S. Carlini**

**co-corresponding author*

Supplementary Methods.

Uncertainty Analysis of Temperature Measurements.

To evaluate the accuracy and performance of temperature sensing circuitry implemented in this study, power supply variations are assessed. As shown in **Supplementary Fig. 6**, the half bridge configuration of NTCs (NTCG063JF103FT, TDK Corporation, Japan) used to measure changes in temperature includes a reference resistor and a 6.5 digit digital multimeter (DMM, USB-4065, National Instruments). **Equation 1** shows the relationship between the resolution of the analog to digital converter (ADC) within the DMM as a function of the output voltage and the supply voltage:

$$ADC\ output = \frac{V_{out}}{V_{ss}} * (2^N - 1) \quad (1)$$

Next, the temperature dependent signal (measurement across the thermistor) or the output voltage (V_{out}) can be seen in Equation 2:

$$V_{out} = \frac{R_{NTC}}{R_{NTC} + R_{REF}} * V_{dd} \quad (2)$$

Combining Equations 1 and 2 yields Equation 3:

$$ADC\ output = \frac{R_{NTC}}{R_{NTC} + R_{REF}} * \frac{V_{dd}}{V_{ss}} * (2^N - 1) \quad (3)$$

Since the reference voltage (V_{ss}) and the supply voltage (V_{DD}) are supplied from the same voltage source, they are effectively identical, which leads to Equation 4:

$$ADC\ output = \frac{R_{NTC}}{R_{NTC} + R_{REF}} * (2^N - 1) \quad (4)$$

Nominal values as well as expected errors for all of the components used in the temperature circuitry at included in **Supplementary Table 3**. Using Equation 2, the partial derivatives of error can be calculated. We find the simplified PDE and calculate that the maximum error contribution of this equipment is <2% at nominal temperature.

$$\sigma V_{out} = \sqrt{\left(\frac{\partial V_{out}}{\partial R_{NTC}}\sigma R_{NTC}\right)^2 + \left(\frac{\partial V_{out}}{\partial R_{REF}}\sigma R_{REF}\right)^2 + \left(\frac{\partial V_{out}}{\partial V_{dd}}\sigma V_{dd}\right)^2}$$

Supplementary Table 1: Nomenclature and definitions.

Nomenclature		
Symbol	Unit	Description
ID	mm	Inner diameter of vessel
OD	mm	Outer diameter of vessel
t	mm	Thickness of vessel wall
h	mm	Depth of skin layer
Q_{Flow}	$mL \cdot min^{-1}$	Volumetric flow rate
k	$W \cdot m^{-1} \cdot K^{-1}$	Thermal conductivity
ΔT	ºC	Temperature change of heater or sensors from baseline values
ΔΔT	ºC	Difference in temperature change during a measurement
PD	$mW \cdot mm^{-2}$	Power density of heater
R_{cond}	$t \cdot m^{2} \cdot K \cdot W^{-1}$	Conductive resistance of tissue
R_{conv}	$ID \cdot m^{2} \cdot K \cdot W^{-1}$	Convective resistance of tissue
Re	unitless	Reynolds number
$R_{contact}$	$mm^{2} \cdot K \cdot W^{-1}$	Contact resistance
Q_{SS}	$mL \cdot min^{-1}$	Flow rate at steady-state heating conditions
τ	s	Time constant, or time at which 63.2% Q_{SS} is reached
**Definitions**		
Symbol	Unit	Description
patent-flow	$mL \cdot min^{-1}$	Healthy vascular flow rate threshold of 600
low-flow	$mL \cdot min^{-1}$	Representative unhealthy vascular flow rate of 100
no-flow	$mL \cdot min^{-1}$	Fully obstructed vascular flow rate of 0

Supplementary Table 2: Previous approaches for VA flow measurements.

Approach	Advantages	Limitations	Reference
Ultrasound	• Wide flow range operation<br>• High flow rate sensitivity (> 180 cm/s)<br>• High accuracy (4% error)<br>• high penetration depth (≥ 25mm)	• bulky form factor of handheld devices<br>• expensive<br>• requires skilled operator<br>• cause localized vascular compression<br>• subject to motion artifacts	[1,2]
Thermodilution	• thorough hemodynamic evaluation, encompassing cardiac output and other relevant indices<br>• allows for calibration of pulse contour analysis, enabling continuous and real-time monitoring of cardiac output	• requires large volume cold bolus injections (increased metabolic burden on patient)<br>• inconvenient for renal failure patients<br>• injection-based<br>• time-consuming<br>• only suitable for critically ill patients	[3]
Plethysmography (PPG)	• measures arterial stiffness, pulse wave velocity, and blood pressure	• low accuracy for flow rate measurements<br>• calculations of flow rate based on AI models from large datasets	[4]
Blood pressure (in dialysis machine)	• assessment of cardiac function and arterial status<br>• monitored during dialysis sessions	• does not calculate flow rates<br>• does not account for vascular dimensions, or blood viscosity<br>• only detects significant occlusions	[5]

Supplementary Table 3: Nominal values and expected errors for components used in temperature circuitry.

Component	Parameter	Value	Unit
Thermistor nominal value (at 25 ºC)	R_{NTC}	10k	Ω
Thermistor tolerance/error	σR_{NTC}	± 1	%
Reference resistor nominal value (at 25 ºC)	R_{REF}	10k	Ω
Reference resistor tolerance/error	σR_{REF}	± 5	%
Supply voltage	V_{DD}	3.3	V
Supply RMS noise	σV_{DD}	1	mV

Supplementary Table 4: Physical and thermal properties of test materials and device components. Each component includes materials considered during *in vitro* or *in vivo* measurements. Unless otherwise stated, values provided through IT IS Foundation or Thermtest Instruments. *Measured experimentally, **provided by manufacturer, ***Reference[6]

Component	Material(s)	Thermal conductivity (k)	Thermal diffusivity (α)	Specific heat capacity	Density	Modulus (E)
	Units	$W\ m^{-1}\ K^{-1}$	$mm^2\ s^{-1}$	$J\ kg^{-1}\ K^{-1}$	$kg\ m^{-3}$	MPa
device	Pyralux	0.26**	0.167**	1089**	1430**	4826**
	3M p2744 adhesive	0.2**	0.139	1258**	1140**	n.d.
fluid	H_2O	0.60 ± 0.01	0.144	4178	994	N/A
	human blood	0.52 ± 0.03	0.137	3617 ± 301	1050 ± 17	N/A
medium	air	0.026 ± 0.00	21.56	1005 ± 0	1.20 ± 0.01	N/A
	SynDaver skin	0.4**	0.10**	3500**	1100**	0.5**
	skin	0.37 ± 0.06	0.10	3391 ± 233	1109 ± 14	0.1
vessel	SynDaver vessel	0.4**	0.10**	3500**	1100**	0.5**
	ePTFE (graft)	0.15 ± 0.01*	0.124	970	3360	31.61 ± 4.76***
	blood vessel	0.46 ± 0.02	calc ~0.126	3306 ± 158	1102 ± 64	N/A

Supplementary Table 5: Literature reports of vascular model properties considered in this study.

Model	Vessel(s)	Vessel Depth (h)	Skin Thermal Conductivity (K)	Vessel Diameter	Blood flow	Reference
Units		mm	W m^{-1} K^{-1}	mm	mL/min	
Human (fistula)	Radial artery / cephalic vein	< 6	0.37 ± 0.06	≥ 6	≥ 600	[7,8]
Human (healthy)	Cephalic vein	2.1 ± 0.8	0.37 ± 0.06	3.12 ± 1.4	28	[9-11]
Porcine (healthy)	Femoral artery	n.d.	0.38 ± 0.01	4 – 6	950 ± 215	[12-14]

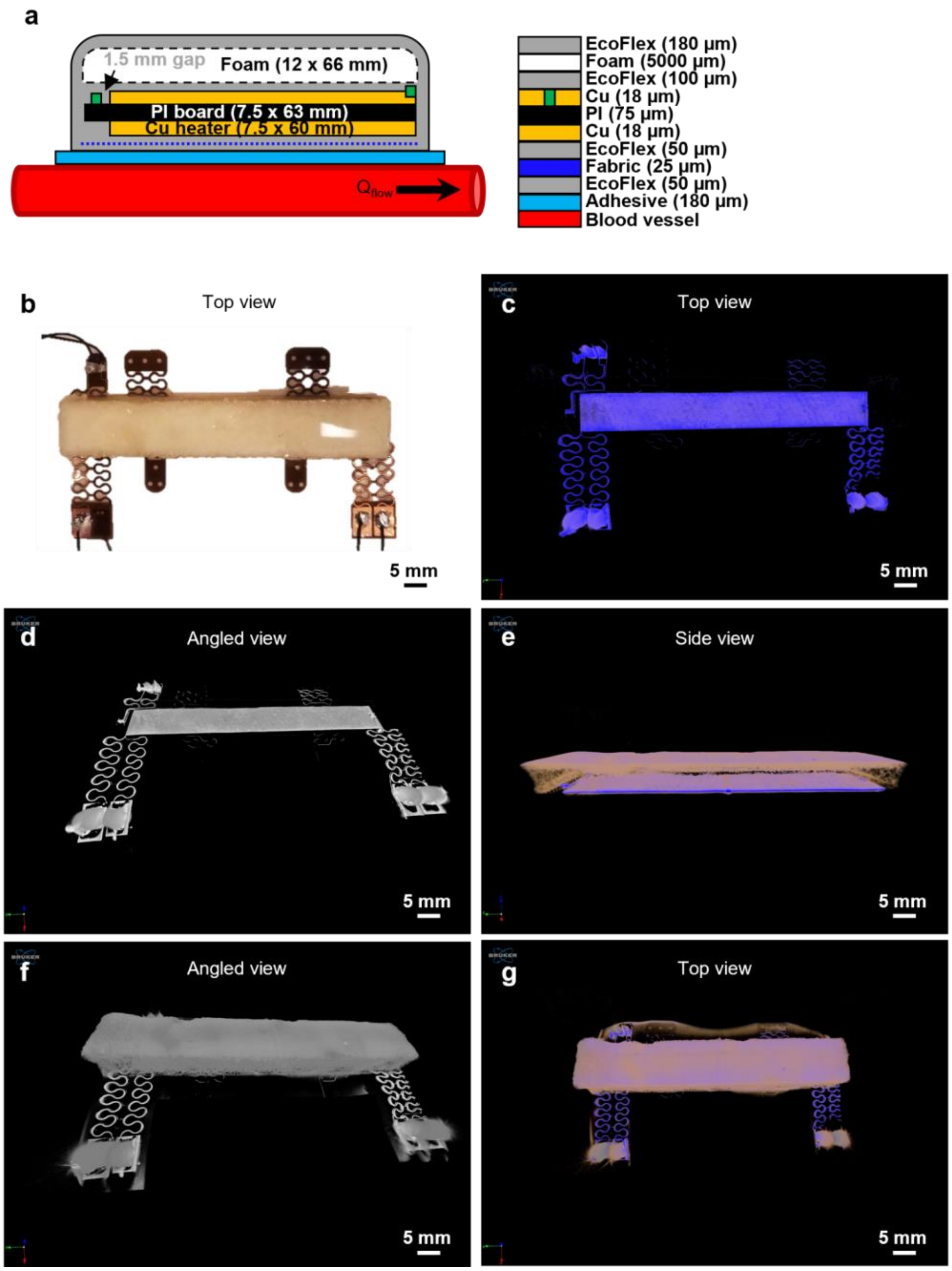

Supplementary Fig 1: **Micro-CT of the encapsulated device for in vivo studies. a**, Schematic of encapsulated devices mounted on a blood vessel. **b**, Camera image of the encapsulated device. **c-g**, Micro-CT scans of encapsulated device depicting the flexible printed circuit board (**c,d**), insulating foam layer (**e**), and composite views (**f,g**).

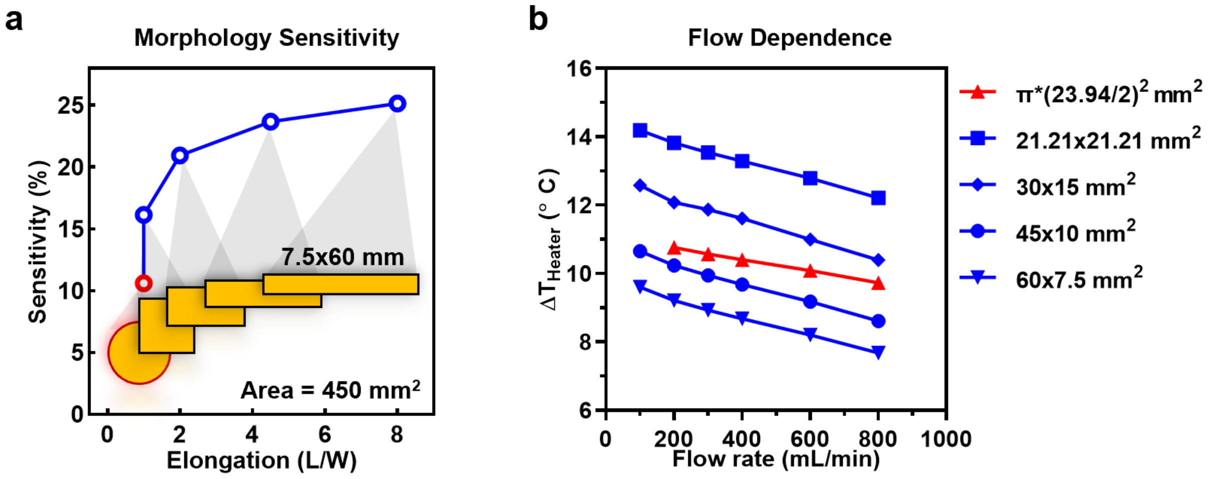

Supplementary Fig. 2: FEA simulations of device morphology. a, Increasing device elongation (L/W), under a conserved heater area of 450 mm^2, increases % sensitivity. Inset diagrams show device shapes. Morphology transition from a circular (red point) to a square (blue points) heater increases sensitivity to flow. **b**, Heater response as a function of flow rate for each morphology.

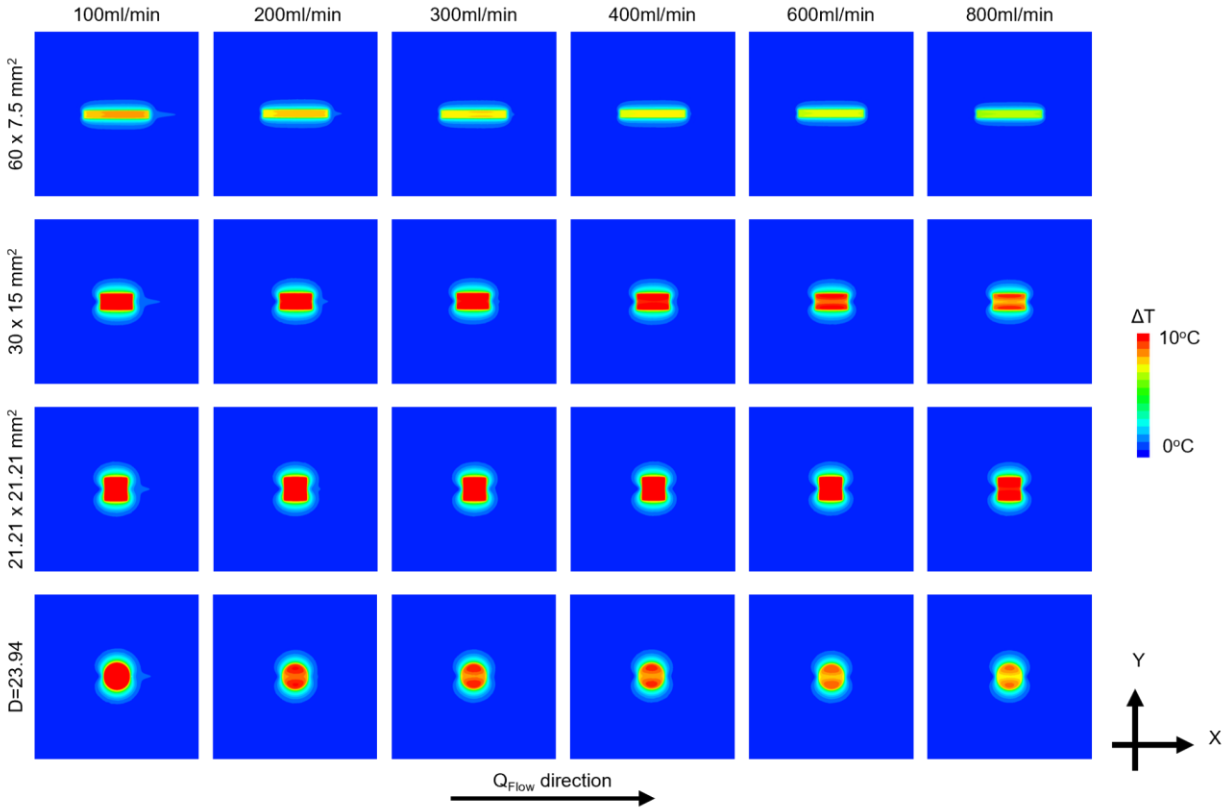

Supplementary Fig. 3: Temperature contours of the top surface of the mounted tissue medium. FEA simulations of different heater morphologies, with conserved surface area (450 mm^2), under variable flow rates (100, 200, 300, 400, 600, and 800 mL/min).

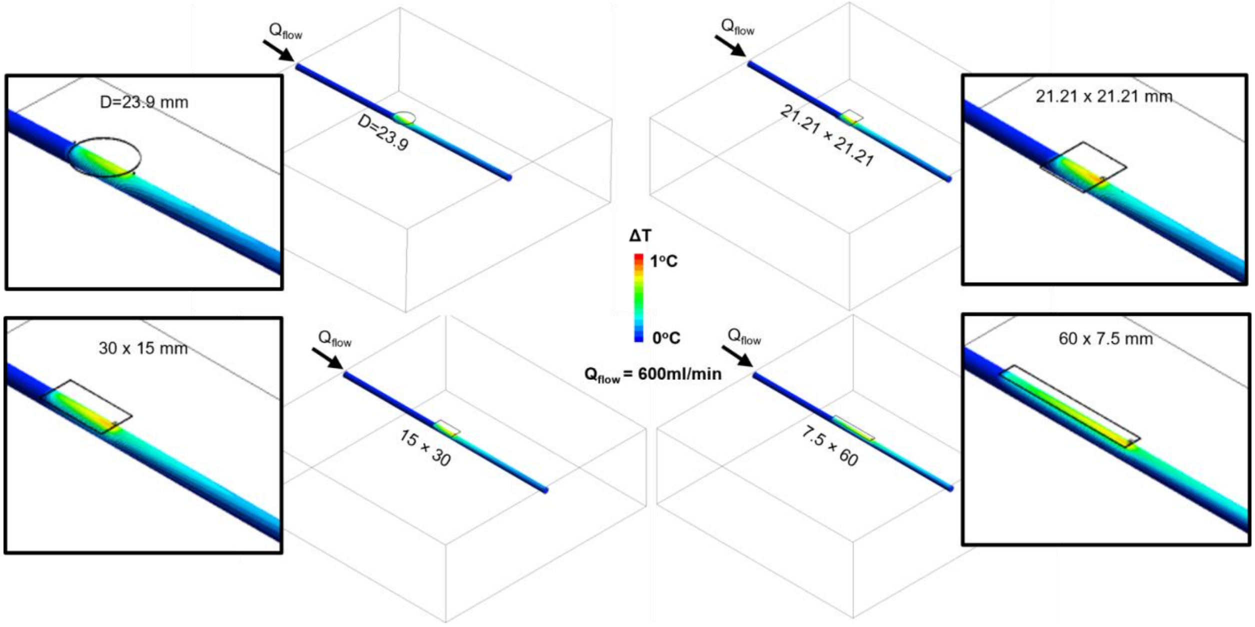

Supplementary Fig. 4: **Temperature contours of the fluid flow from mounted devices**. FEA simulations of different heater morphologies, with conserved surface area (450 mm^2), under high flow (600 mL/min). Outline of the tissue medium box and device are shown.

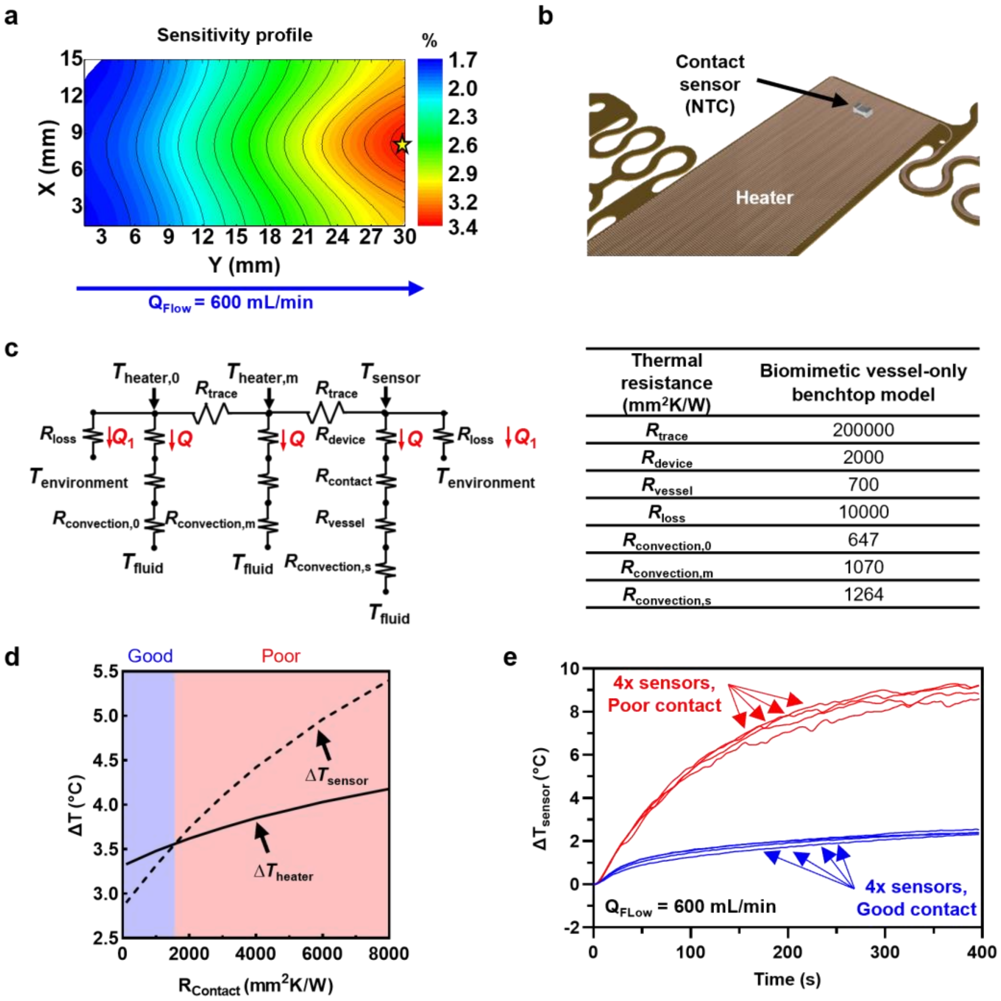

Thermal resistance (mm^2K/W)	Biomimetic vessel-only benchtop model
R_{trace}	200000
R_{device}	2000
R_{vessel}	700
R_{loss}	10000
$R_{convection,0}$	647
$R_{convection,m}$	1070
$R_{convection,s}$	1264

Supplementary Fig. 5: FEA simulations of the device sensitivity. **a**, Contour map of % sensitivity along the surface of a heater (30 x 15 mm^2) mounted on a tissue-embedded rigid vessel. The highest sensitivity along the heater occurs at the lengthwise edge downstream to the direction of flow (Q_{Flow}). The star symbol denotes the contact sensor's location for measuring contact resistance. **b**, Placement of negative temperature coefficient (NTC) contact sensor at the downstream position on the heater surface. **c**, Thermal equivalent model (left) of the device on a biomimetic vessel. Thermal resistance values are calculated based on experiments and shown in the table (right). The device is powered at 1mW/mm^2. The heater temperature is the device's average temperature which is $T_{heater} = (T_{heater,0} + T_{heater,m} + T_{sensor})/3$. **d**, Temperature comparison of the heater vs. contact sensor as a function of device-tissue contact quality. The contact sensor shows a higher dependence on tissue contact, whereas the heater tolerates heterogenous contact issues better. **e**, Thermal measurements from the device contact sensor from four devices during 400s heating when mounted on graft + biomimetic skin at 600 mL/min. Increased temperature rise and noise for sensors with poor contact illustrate increased thermal impedance at the skin-device interface and air convection, respectively.

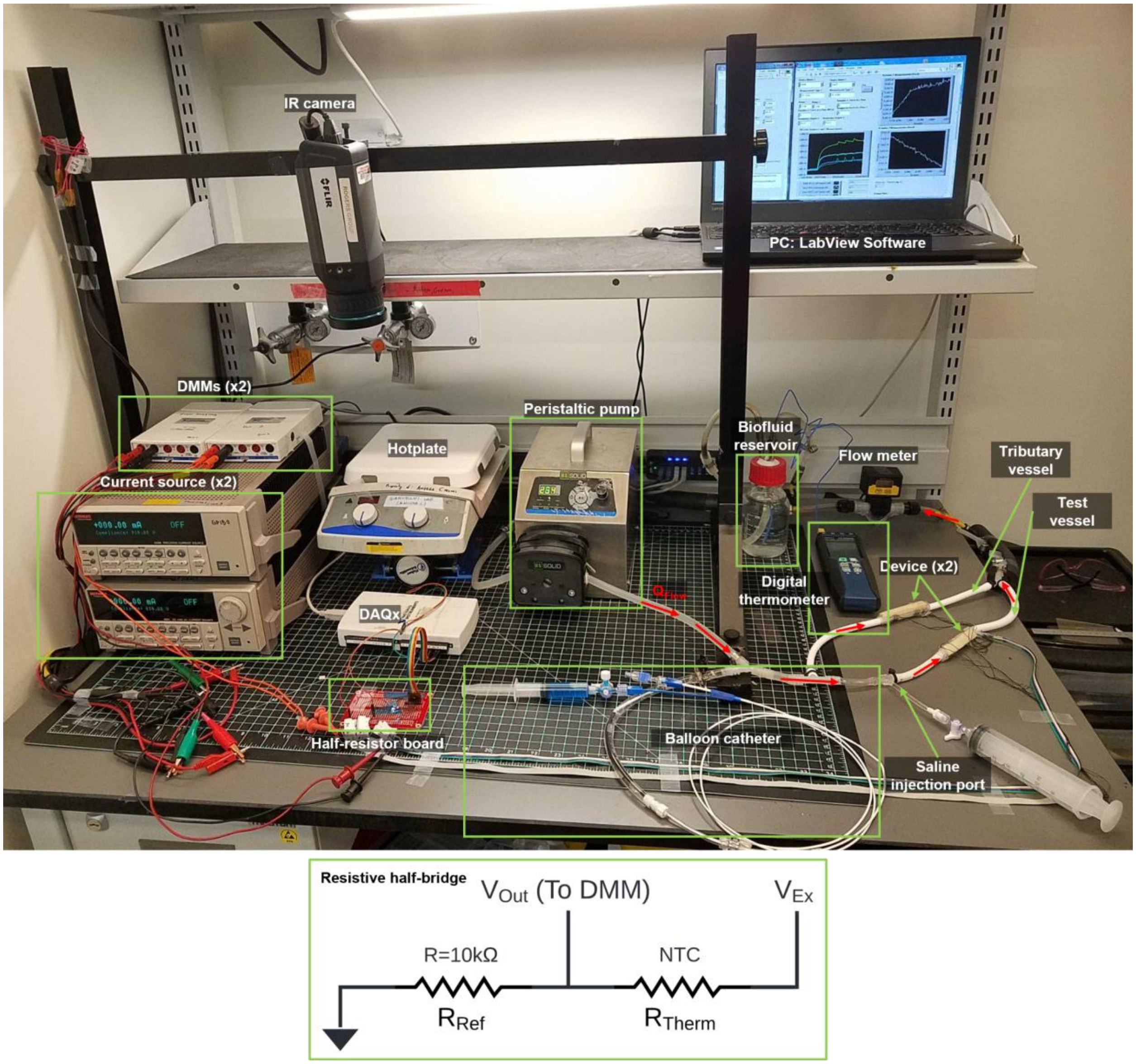

Supplementary Fig 6: Benchtop setup for two-device operation. Inset image (below) showing the circuit for the resistive half-bridge for negative temperature coefficient (NTC) sensor measurements.

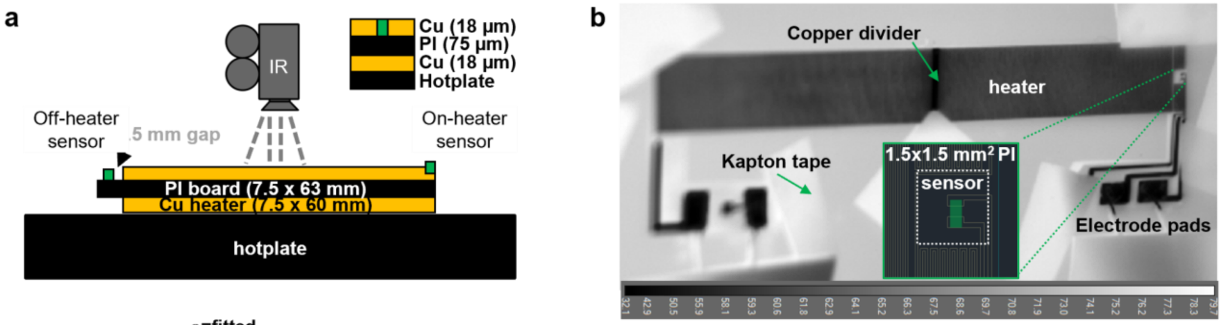

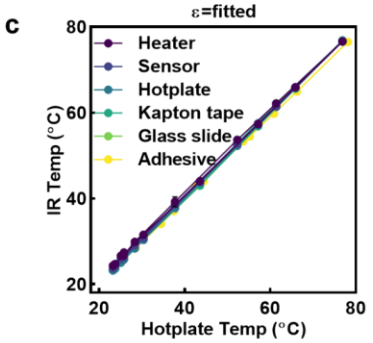

d

Component	Composition	Fitted ε	Lit. ε
Heater	PI/Cu (54:46%)*	0.618	n/a
Sensor	PI/Cu/resistor	0.773	n/a
Hotplate	white ceramic	0.91	0.9-0.95
Kapton tape	PI	0.973	~0.95
Copper divider	Cu (laser cut)	0.204	0.05-0.78
Glass slide	SiO_2	0.876	0.85-0.95
Adhesive	Silicone (top)	0.93	~0.9-1.0

*Calculated ε_{Heater} agrees with ~54:46% PI/Cu on surface

Supplementary Fig. 7: Calibration of IR camera. a, Schematic of an unencapsulated device mounted on a hotplate for heater IR emissivity calibrations. **b**, IR image of the device with labeled components. Magnified view of the sensor on PI pad (1.5x1.5 mm^2). **c**,**d**, Measured IR temperature using fitted emissivity values for each material, plotted as a function of hotplate temperature measured by a thermocouple. Table (**d**) summarizes fitted and literature emissivity (ε) values for each component analyzed by IR. Heater composition of PI and Cu are calculated using fits for the copper divider and Kapton tape.

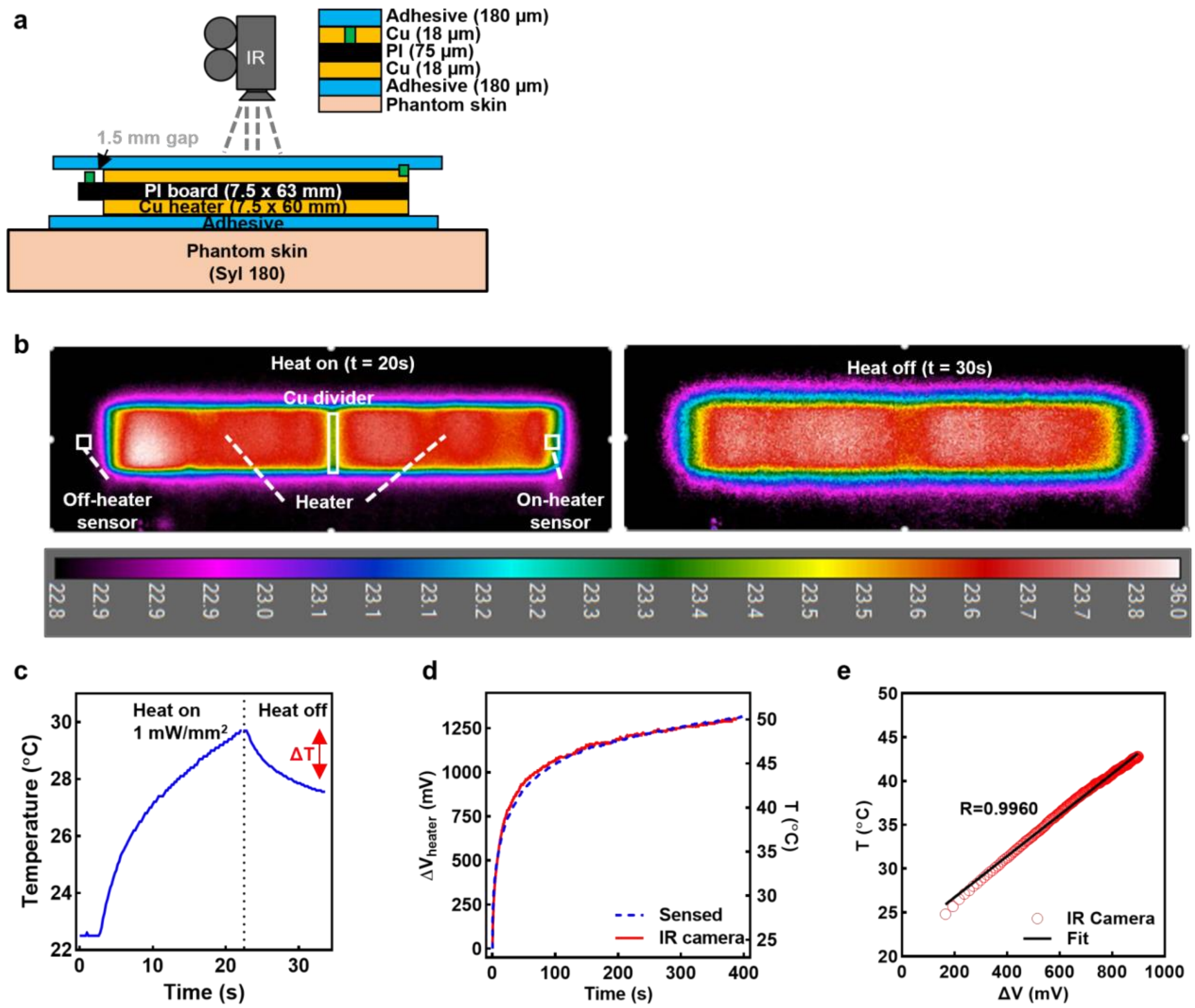

Supplementary Fig. 8: Correlation of IR temperature with sensed heater voltage. **a**, Schematic of device adhered to a phantom skin hotplate for IR and sensed measurements. **b**,**c**, IR images (**b**) of device with labeled components at t = 20 and 30s of heating at 1 mW/mm^2, and corresponding IR temperatures (°C) plotted as a function of time. **d**, Sensed heater voltage and IR camera temperature as a function of time. **e**, Conversion and fit (R=0.9960) of sensed heater voltage to temperature via calibration with IR temperature showing experimentally achieved resolution and goodness of fit. R=0.9960.

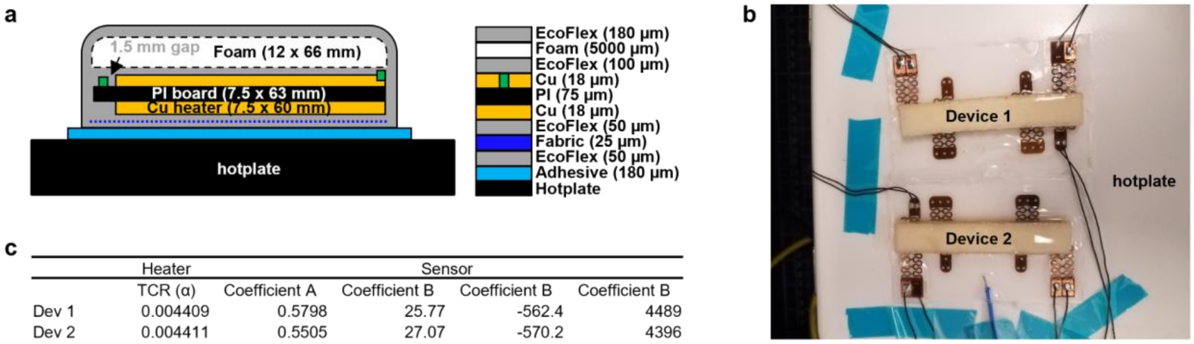

c

	Heater	Sensor			
	TCR (α)	Coefficient A	Coefficient B	Coefficient B	Coefficient B
Dev 1	0.004409	0.5798	25.77	-562.4	4489
Dev 2	0.004411	0.5505	27.07	-570.2	4396
Dev 3	0.004108	0.5939	25.87	-614.3	5477
Dev 4	0.004316	0.5974	23.08	-453.2	3173
Dev 5	0.004161	0.5831	26.16	-594	4986
Dev 6	0.004285	0.557	26.42	-561.3	4407
Literature	0.00393	0.5738	25.3	-521.9	3888

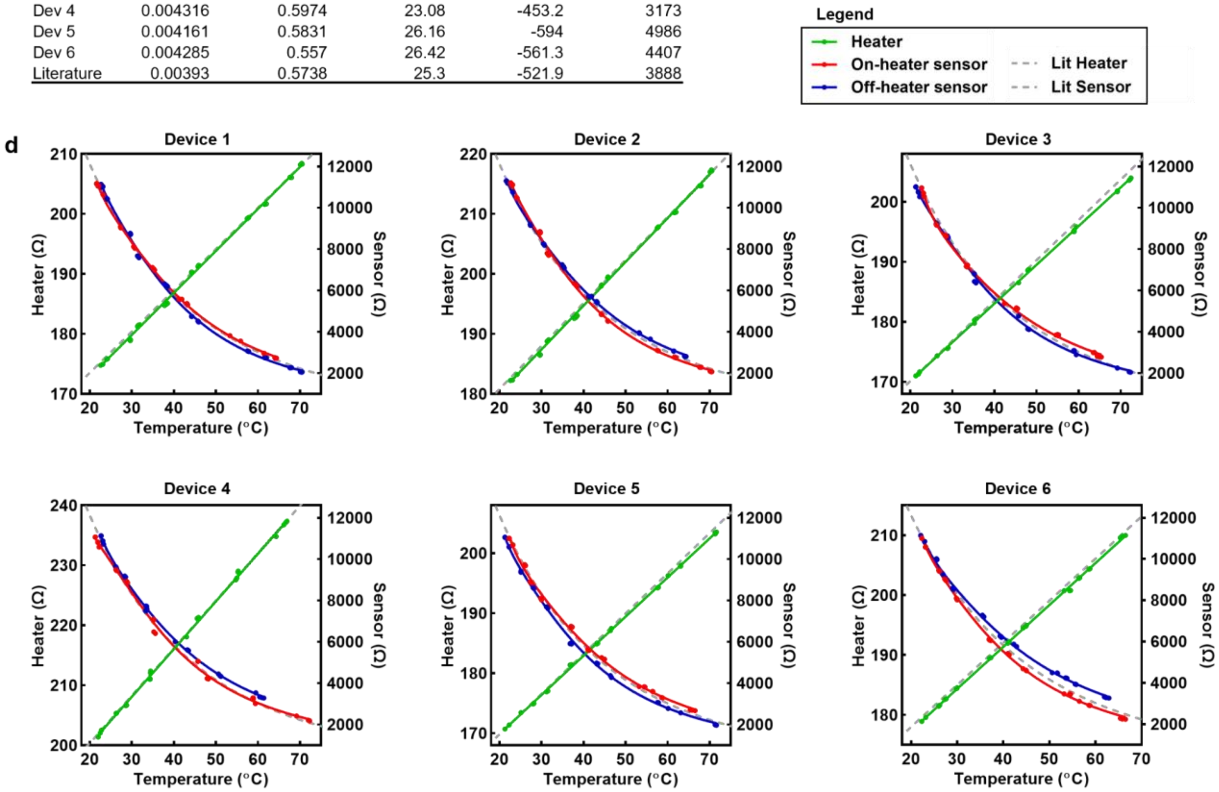

Supplementary Fig. 9: Device Calibration. **a,** Schematic of encapsulated devices mounted on a hotplate for calibration. **b**, Corresponding picture. **c**, Table summarizing calculated temperature coefficient of resistance (TCR) for the heater and Steinhart-Hart equation coefficients for six devices, along with literature values **d**, Resistance of heater or sensor as a function of hotplate temperature. Hotplate temperatures are verified by IR thermography and thermocouple measurements.

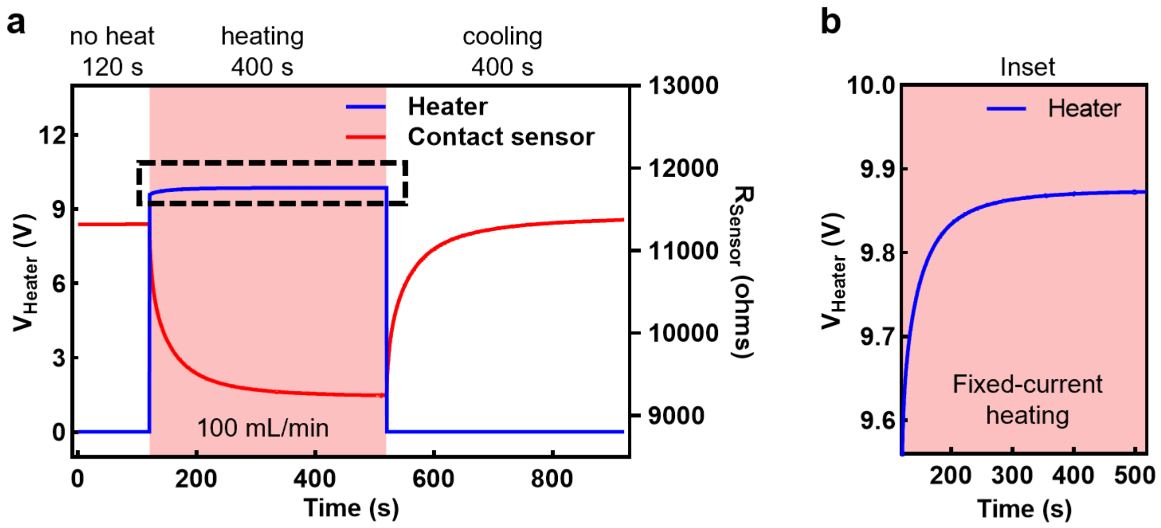

Supplementary Fig. 10: Representative thermal sensing protocol. **a**, Measurements of heater voltage and sensor resistance over an initial no heating step (100 µA, 120 s), heating step (PD 1 mW/mm^2, 400 s), and cooling step (100 µA, 400 s). Q_{Flow} = 100 mL/min. **b**, Inset showing heater voltage change due to joule heating at fixed current (~47 mA). Baseline temperature 21ºC.

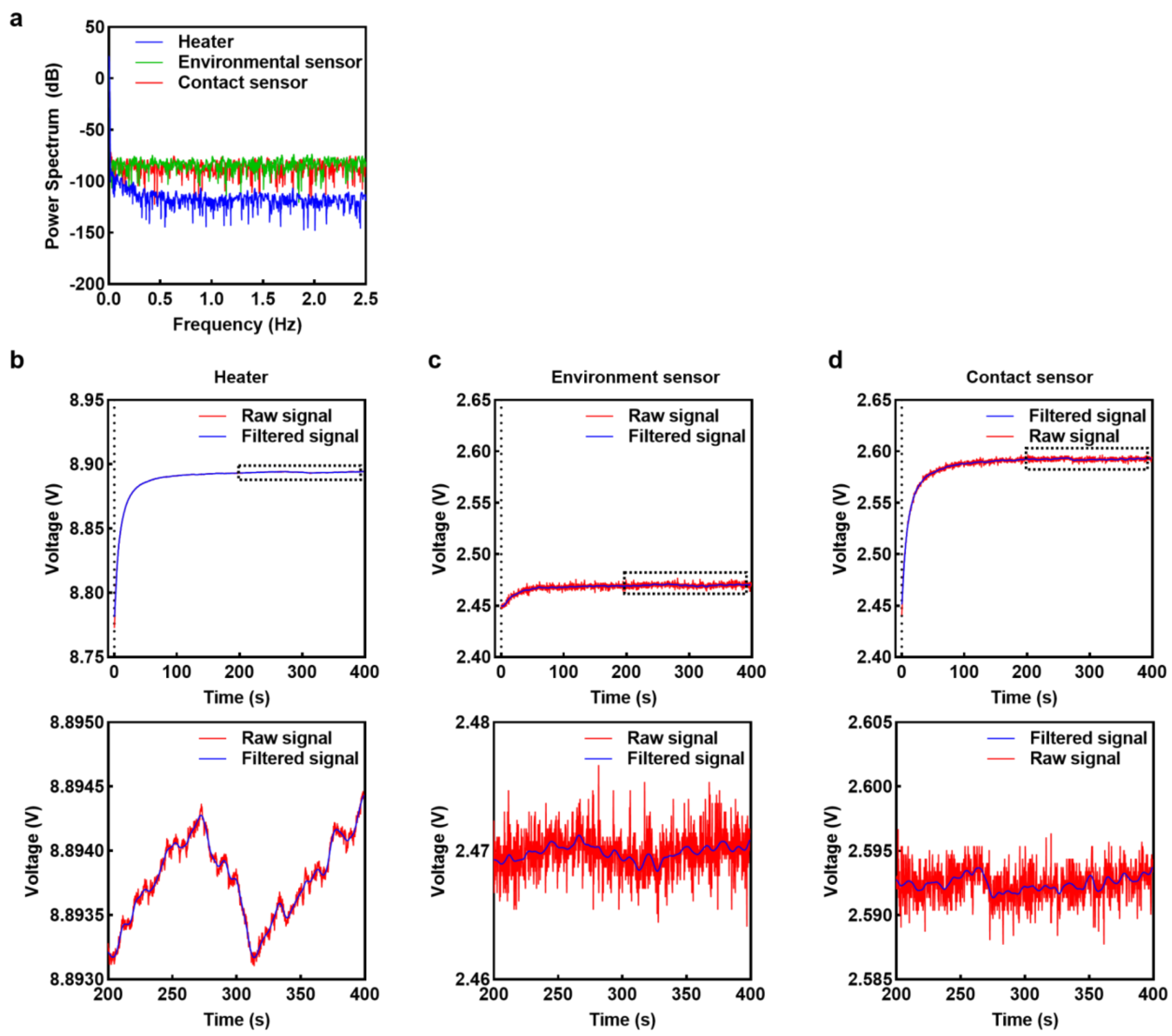

Supplementary Fig. 11. Signal filtering. a, Representative power spectral density for the heater, environmental sensor, and contact sensor. **b**, Voltage as a function of time for the heater showing raw and filtered signal (top). Inset box is magnified (bottom). **c**,**d**, Corresponding plots for the environmental sensor (**c**) and contact sensor (**d**).

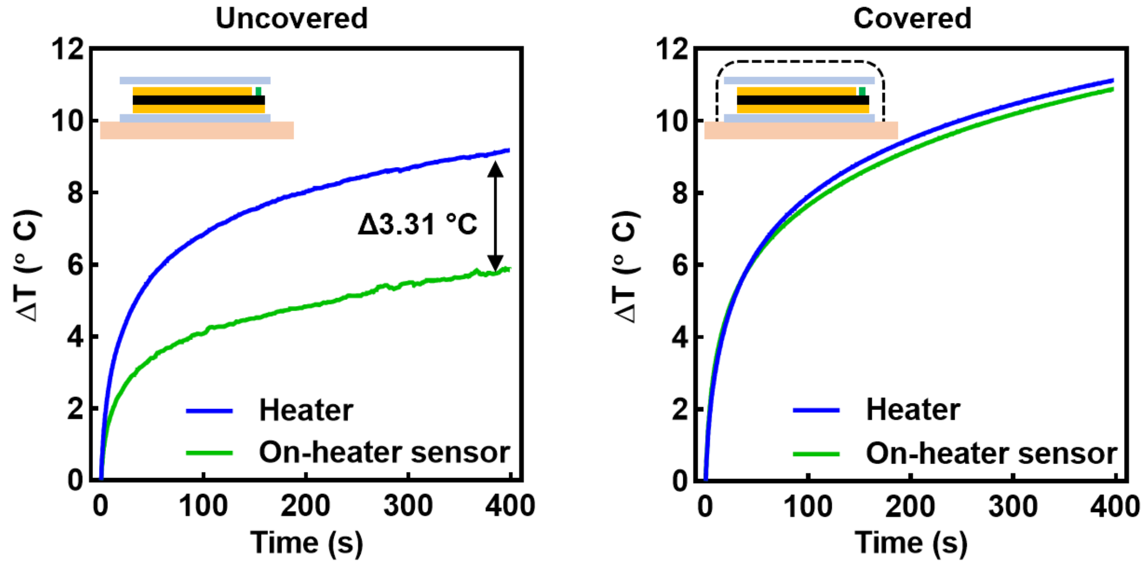

Supplementary Fig. 12: Thermal insulation to minimize convective cooling and noise. Temperature change of heater and contact sensor as a function of time. Inset shows a schematic of the device mounted on phantom skin without (left) or with (right) thermally insulating foam layer. Convective cooling of the uncovered device leads to significantly lower sensor ΔT and noise. Q_{Flow} = 0 mL/min. Device PD 1 mW/mm^2 (n=1). Baseline temperature 21ºC.

a

b

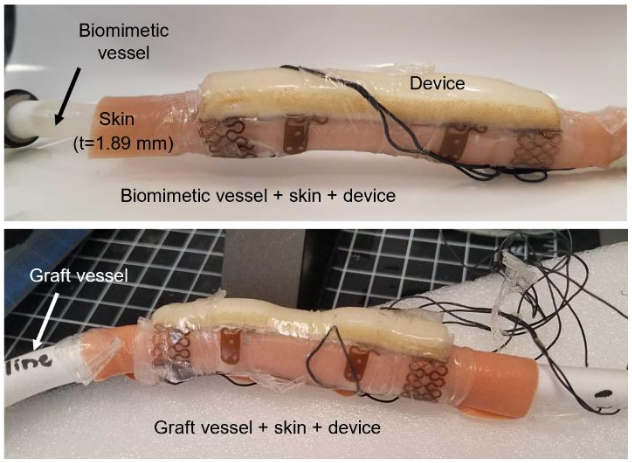

c

	OD (mm)	ID (mm)	t_{vessel} (mm)	t_{skin} (mm)
Biomimetic	6.65	5.95	0.35	1.89
Graft	8.54	6.08	1.13	1.89

Supplementary Fig. 13: Benchtop models consist of a physiologically vessel with a mounted biomimetic skin layer. Images of the device mounted on (a) biomimetic and (b) graft vessels with an underlying layer of skin. **c**, Table of geometric parameters for each phantom model.

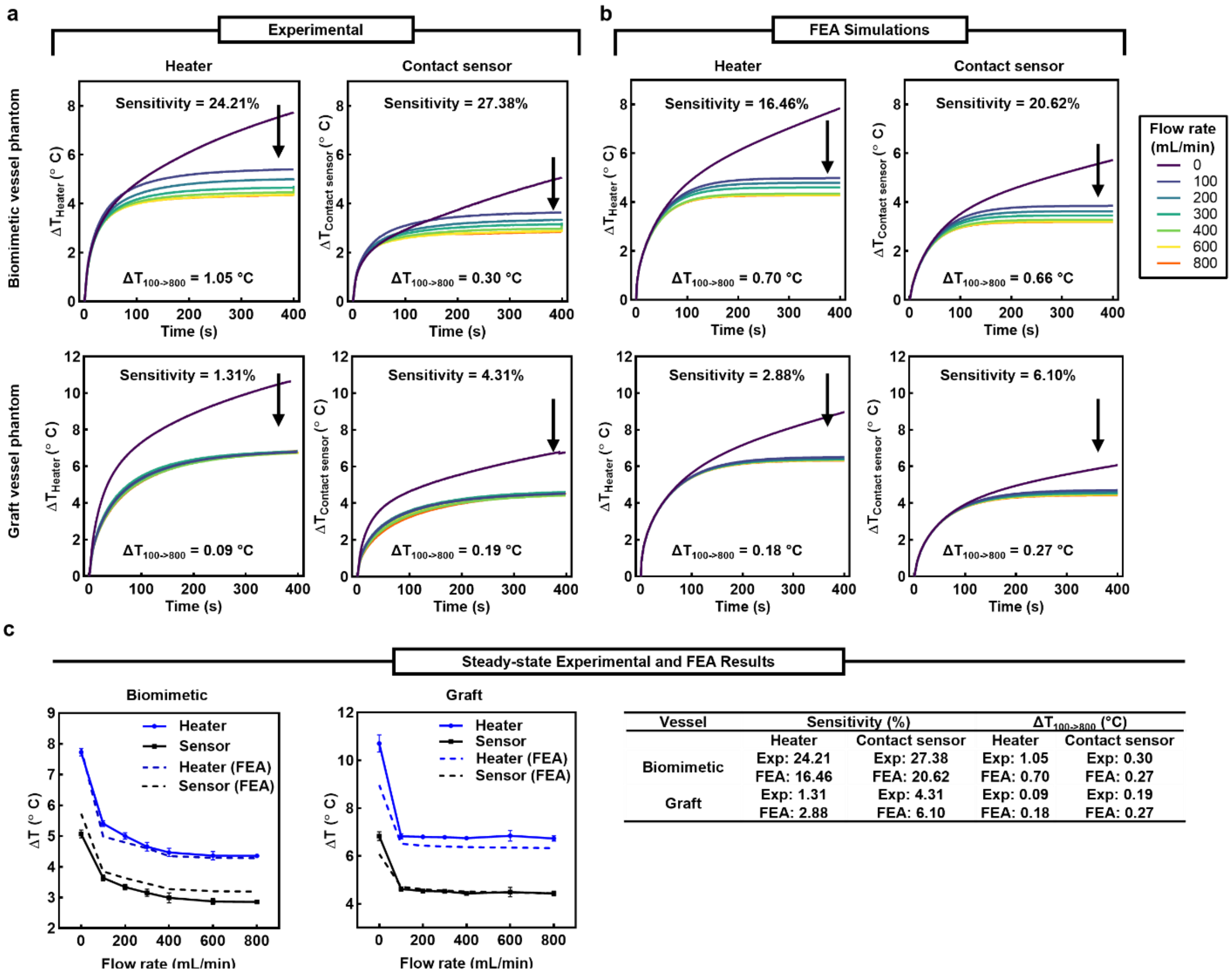

Vessel	Sensitivity (%)		$\Delta T_{100\text{->}800}$ (°C)	
	Heater	Contact sensor	Heater	Contact sensor
Biomimetic	Exp: 24.21<br>FEA: 16.46	Exp: 27.38<br>FEA: 20.62	Exp: 1.05<br>FEA: 0.70	Exp: 0.30<br>FEA: 0.27
Graft	Exp: 1.31<br>FEA: 2.88	Exp: 4.31<br>FEA: 6.10	Exp: 0.09<br>FEA: 0.18	Exp: 0.19<br>FEA: 0.27

Supplementary Fig. 14: Experimental and FEA simulations of thermal flow sensing on benchtop vascular phantoms. a-d, Temperature changes for the device heater (left) and contact sensor (right) as a function of time at Q_{Flow} = 0, 100, 200, 300, 400, 600, and 800 mL/min. **a**, Data collected using the biomimetic (top) and graft (bottom) vessel phantoms correspond to **Fig. 3c,d**. Arrows depict an increasing flow rate. Sensitivity calculated according to **Equation 1**. **b**, Matching FEA simulations. **c**, Steady-state temperature changes (t = 400s) as a function of flow rate, depicting experimental (solid lines) and FEA simulations (dashed lines) for the device heater (blue) and contact sensor (black). Corresponding table (right) of sensitivity and relative temperature values. Device powered at 1 mW/mm^2. (n = 4). Baseline temperature 21ºC.

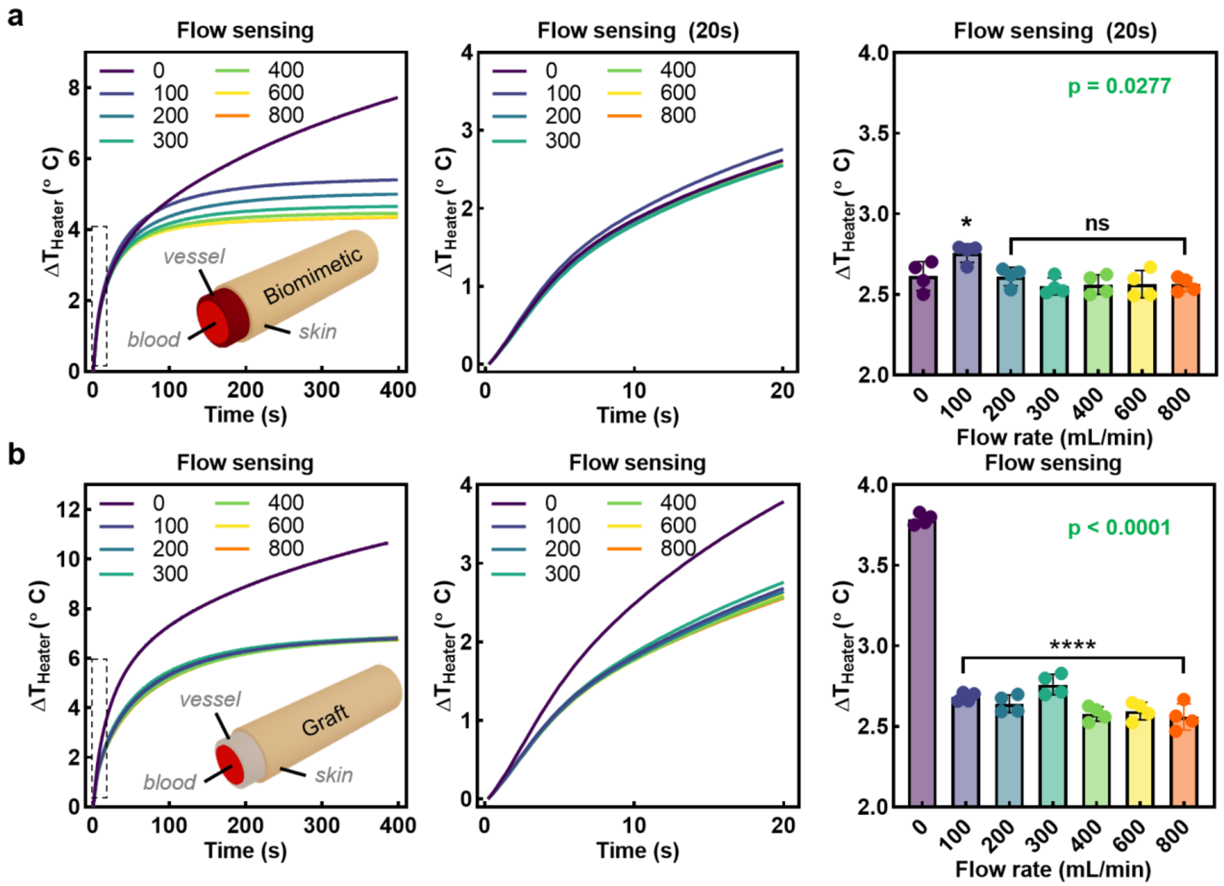

Supplementary Fig. 15: Decoupled impact of flow convection and vessel wall conduction on different vascular phantoms. a, Steady-state flow sensing for 400s on a biomimetic vessel (left). Inset box highlights transient kinetics in the first 20 s (middle) of heating, at which vessel wall conduction predominates. Heater ΔT at 20 s (right) for the high *k*, low thickness (t) vessel shows minimal thermal impedance. **b**, Corresponding data on a graft vessel. Under *no-flow* conditions, thermal impedance from the low *k*, high t vessel predominates. Combined, initial kinetics under *no-flow* conditions can help delineate and characterize the type of vascular access (VA) being analyzed as either a fistula or synthetic graft. Device powered at 1 mW/mm^2. (n = 4). Baseline temperature 21ºC.

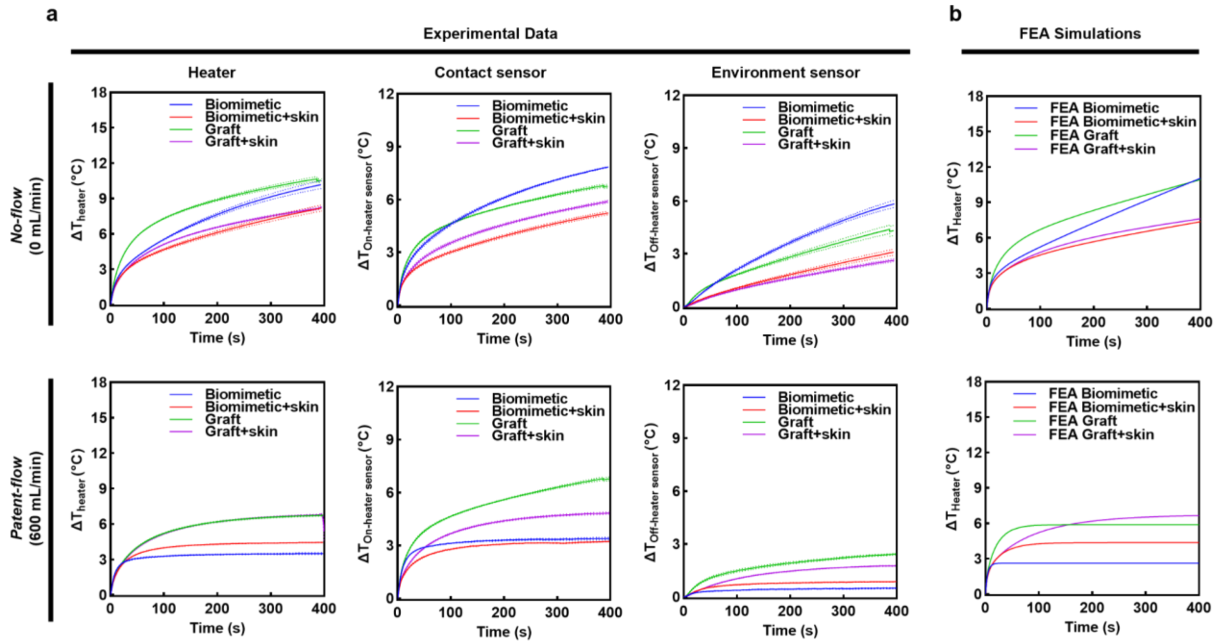

Supplementary Fig. 16: Influence of 1.89mm skin layer. a, Temperature change as a function of time for the device heater (left), contact sensor (middle), and environment sensor (right) under *no-flow* (0 mL/min, top) and *patent-flow* (600 mL/min, bottom) conditions. Measurements were conducted on biomimetic and graft vessels, with and without a skin layer (h=1.89 mm). **b**, Corresponding FEA simulations. Data corresponds to **Fig.3f**. Device powered at 1 mW/mm^2. (n = 4, mean ± SEM). Baseline temperature 21ºC.

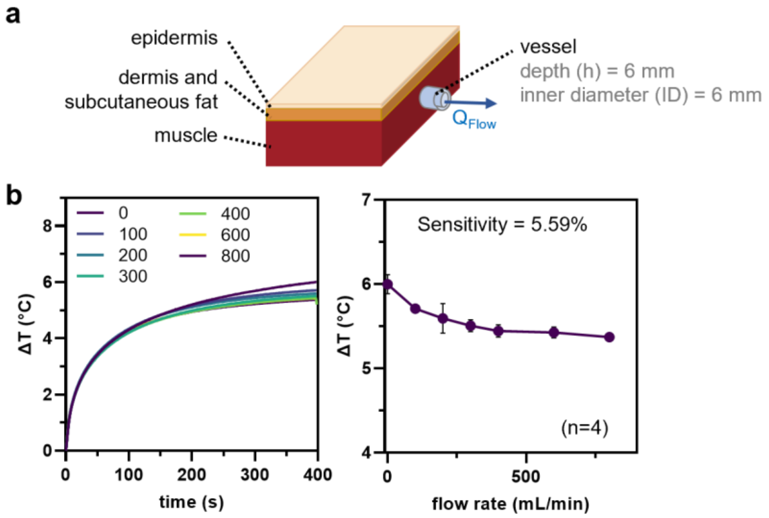

Supplementary Fig. 17: Influence of increased depth and tissue heterogeneity. a, Image of biomimetic vessel pad consisting of 6mm diameter vessels embedded 3mm below the heterogenous tissue surface (epidermis, dermis, subcutaneous fat) and above a muscle tissue layer. **b**, Temperature change as a function of time at different flow rates (0, 100, 200, 300, 400, 60, 800 mL/min) for the device heater and corresponding steady state values. Sensitivity is 5.59% (compares with 24.21% for vessel with 1.89mm skin layer in **Supplementary Fig. 14**). Device powered at 1 mW/mm^2. (n = 4, mean ± SEM). Baseline temperature 21ºC.

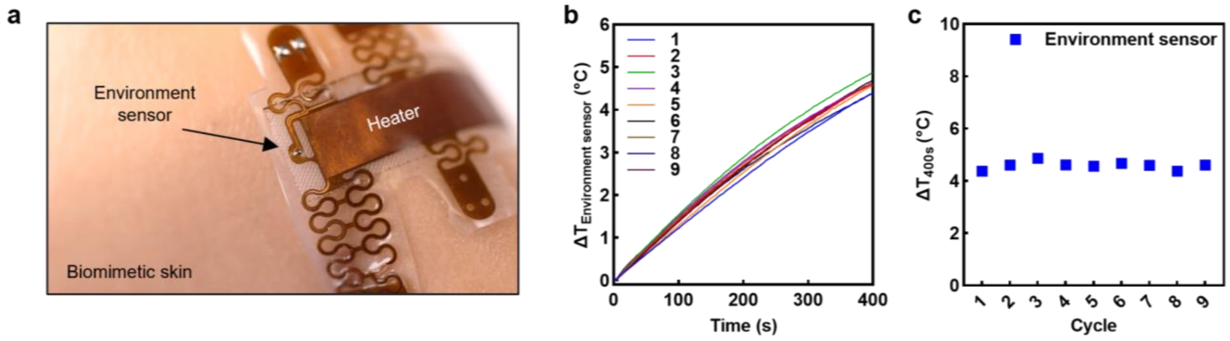

Supplementary Fig. 18: Influence of repeat heating on biomimetic tissue dehydration. **a**, Picture of biomimetic skin containing physiologically relevant moisture content to adult human skin, with mounted wearable flow sensor (no foam for visualization). **b**, Thermal measurements of the device (foam incorporated) depicting environment sensor temperature during repeat cycles of 400s heat-on, followed by 400s heat-off. **c**, Corresponding signal at t=400s heating for each cycle. (n=9). Baseline temperature 21ºC.

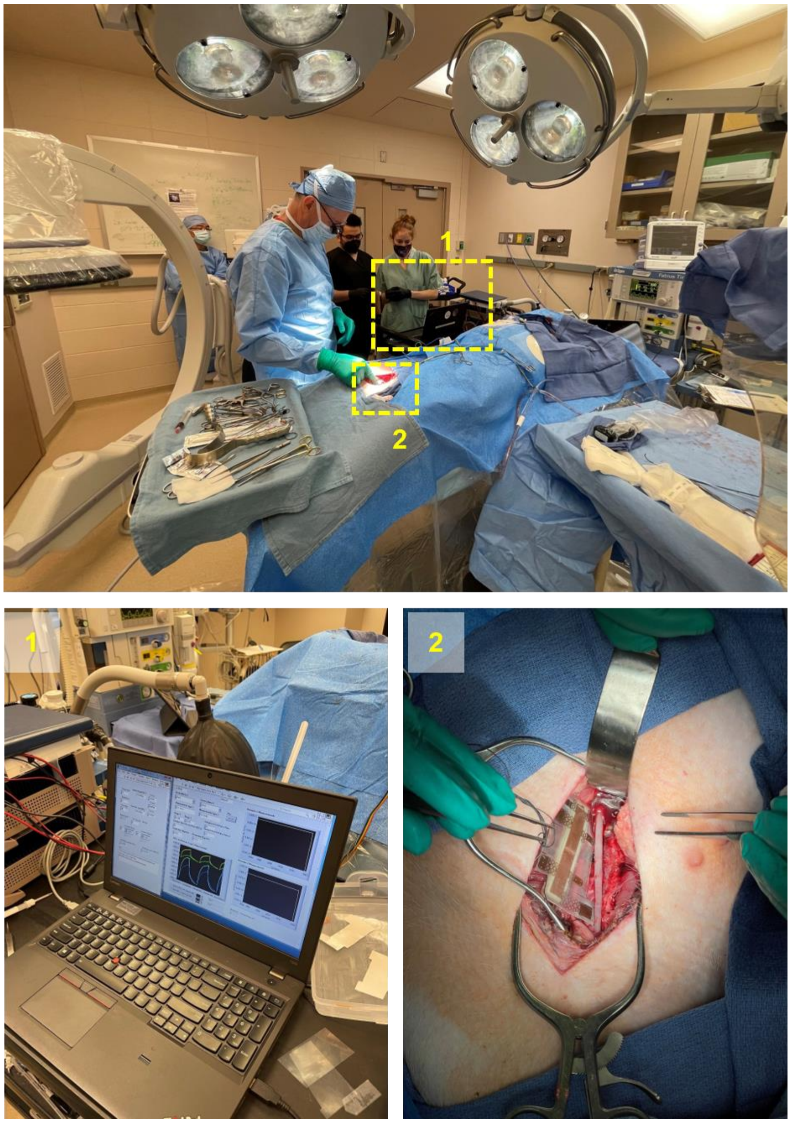

Supplementary Fig. 19: Images of the surgical suite during large animal model tests.

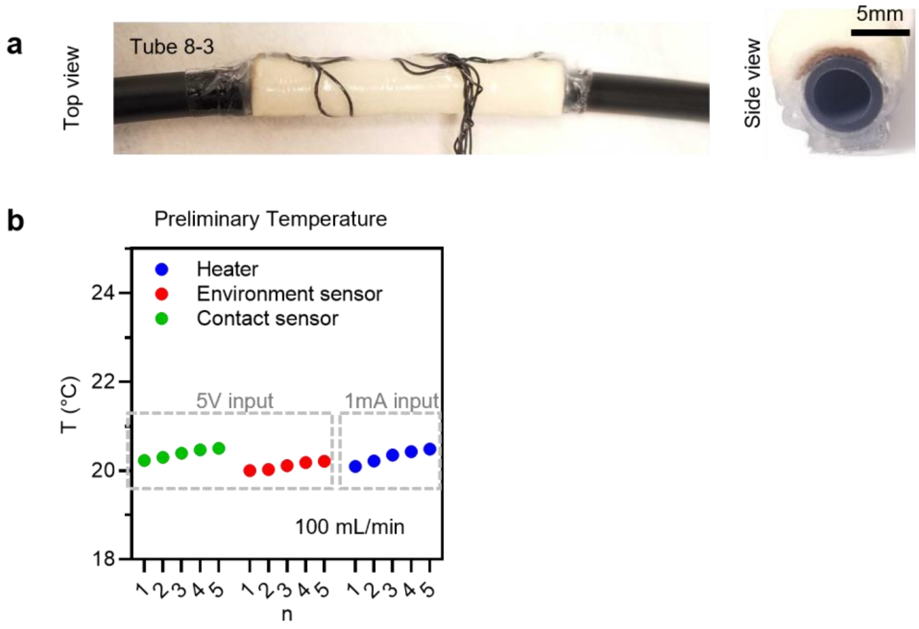

Supplementary Fig. 20: Response to mechanical manipulation via bending. a, Camera images of manually bent device conformed to rigid, conductive PTFE, benchtop vessel surface. Side view shows the bending of the device around the vessel surface. **b**, Temperatures measured by device components before repeat thermal actuation experiments (n=5), demonstrating negligible drift in calibrations at room temperature (20.5 °C by a thermocouple). Note voltage and current inputs for the heater and sensors.

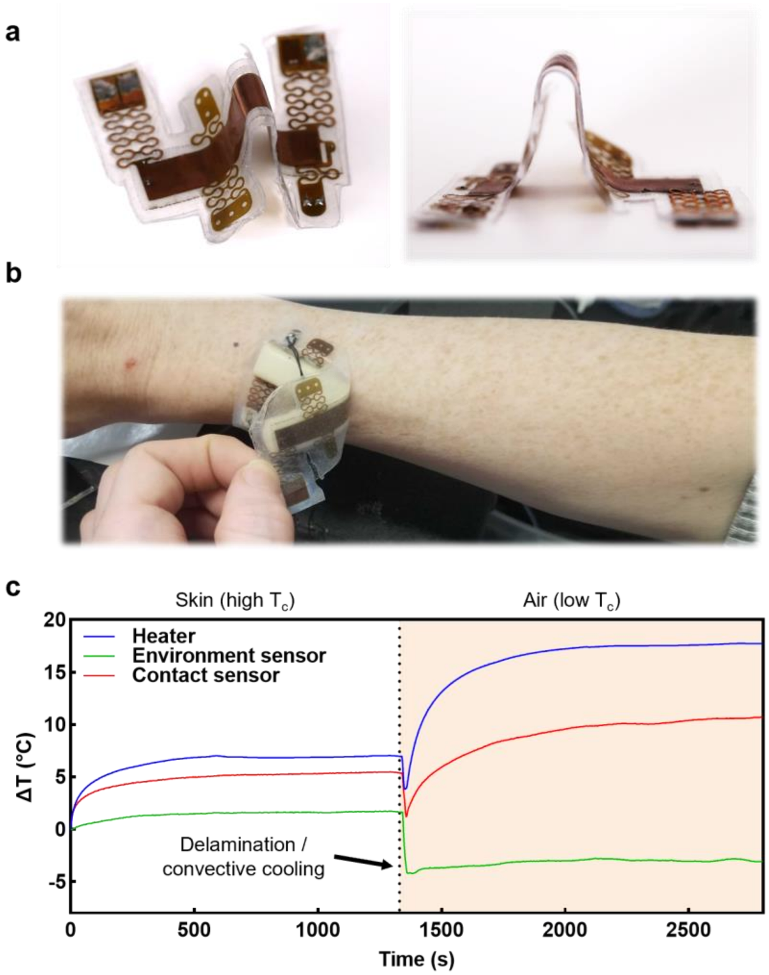

Supplementary Fig. 21: Response to mechanical manipulation via folding. a, Camera images of manually folded devices. **b**, Image of manual delamination of device from skin. **c**, Real-time thermal sensing on the skin, during manual delamination, and under no contact (air). Convective cooling was observed during delamination and exposure to air. Note that mechanical manipulation does not interfere with device operation or cause signal dropouts. Baseline skin temperature is 32 ºC and air temperature is 21 ºC.

a

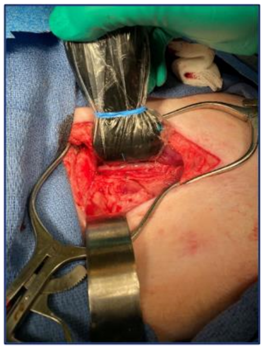

b

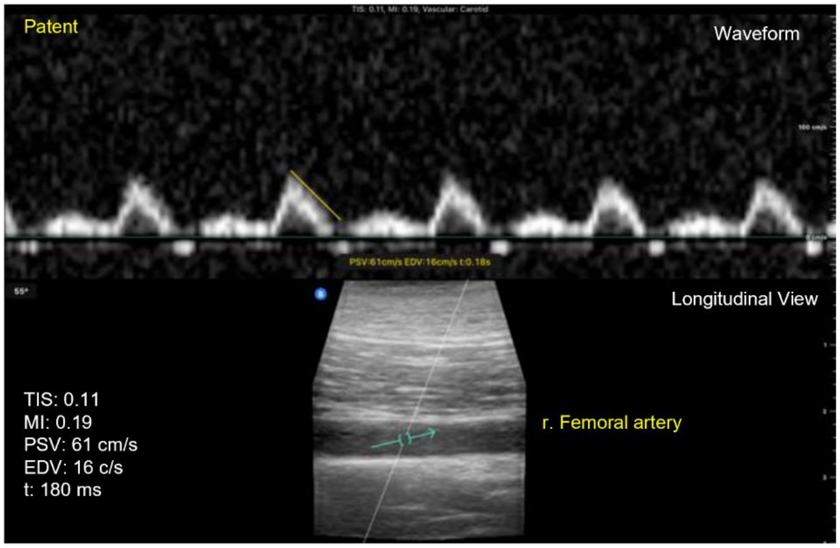

c

Location	Pig #	t (µm)	OD (µm)	ID (µm)	PSV (cm/s)	EDV (cm/s)	US Flow (mL/min)
Neck skin	1	n/a	n/a	n/a		n/a	
r. Femoral artery	1	183	5733	5367	61	16	828
e. Jugular vein	2	321	3738	3096	70	n.d.	316
r. Femoral artery	2	425	5815	4965	74	17	860
Chest skin	2	n/a	n/a	n/a		n/a	
l. Femoral artery	2 (cntr)	551	4225	3123	70	n.d.	322

Supplementary Fig. 22: In vivo tissue analysis and duplex ultrasound (US). a, Image of ultrasound (US, Butterfly iQ+) probe over r. femoral artery. **b,** Corresponding US waveform (top) and longitudinal view (bottom). Labels for TIS (thermal index for soft tissue), MI (mechanical index), PSV (peak systolic velocity), EDV (end diastolic velocity), and t (pulse time). The y-axis displays a vessel depth of 20 mm. **c**, Table summarizing the tissue locations, vascular dimensions (measured from histology), and US flow velocities (measured by US) and volumetric rates (calculated from histology and PSV). Note that data for pig 2 r. femoral artery is displayed in main text **Fig. 4**.

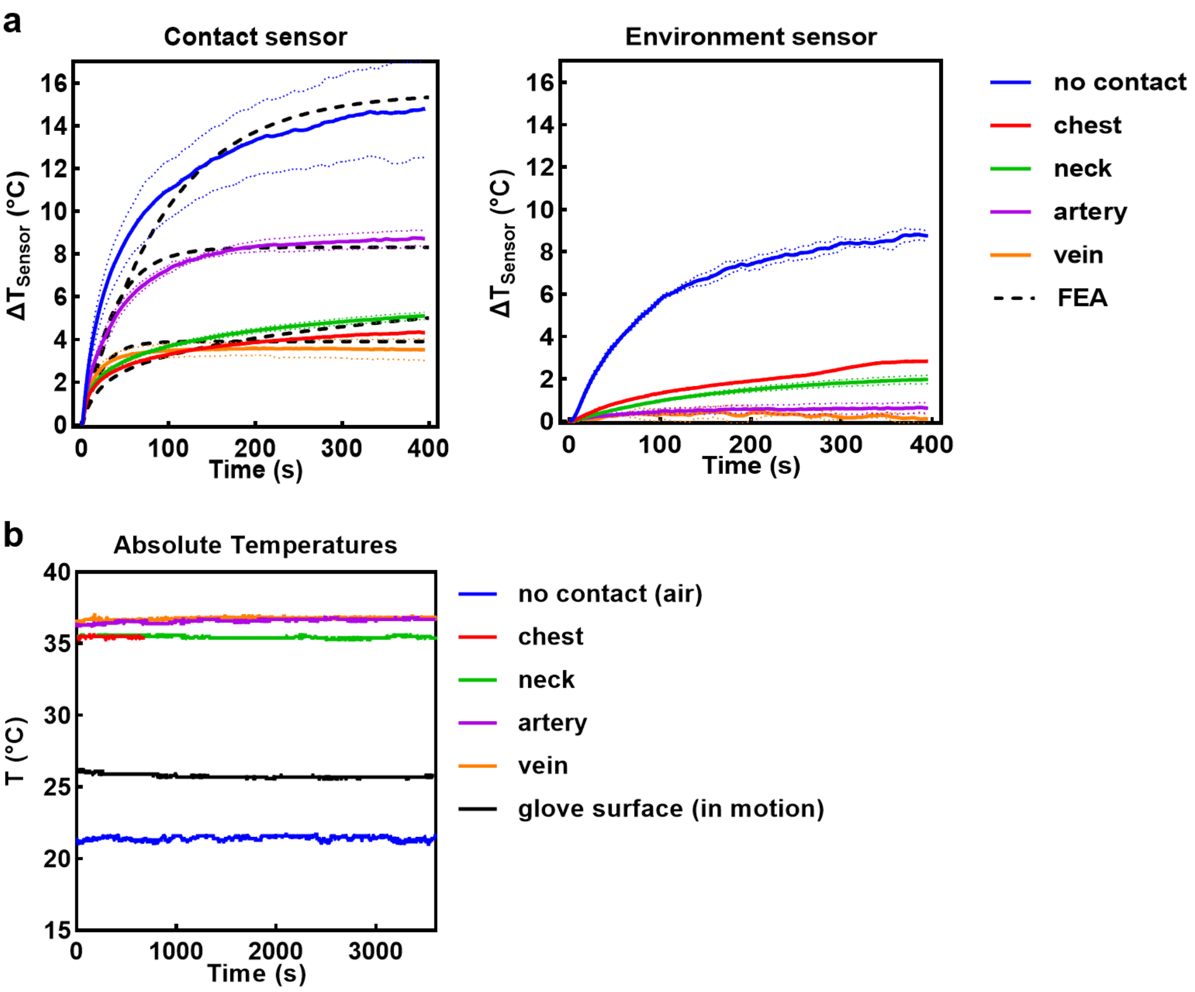

Supplementary Fig. 23: In vivo tissue measurements in a large animal model. a, Temperature sensing time course measurements from the contact sensor (left) and environment sensor (right). **b**, Continuous thermocouple measurements of absolute temperatures on various tissue surfaces ~5-10 cm away from the heater during device measurements. Glove surface corresponds to a device adhered to the surface of a surgical glove worn by the operator during each measurement and manipulation. Data corresponds to main text **Fig. 4c**. Device operation at 1 mW/mm^2 (n = 3-4, mean ± SD).

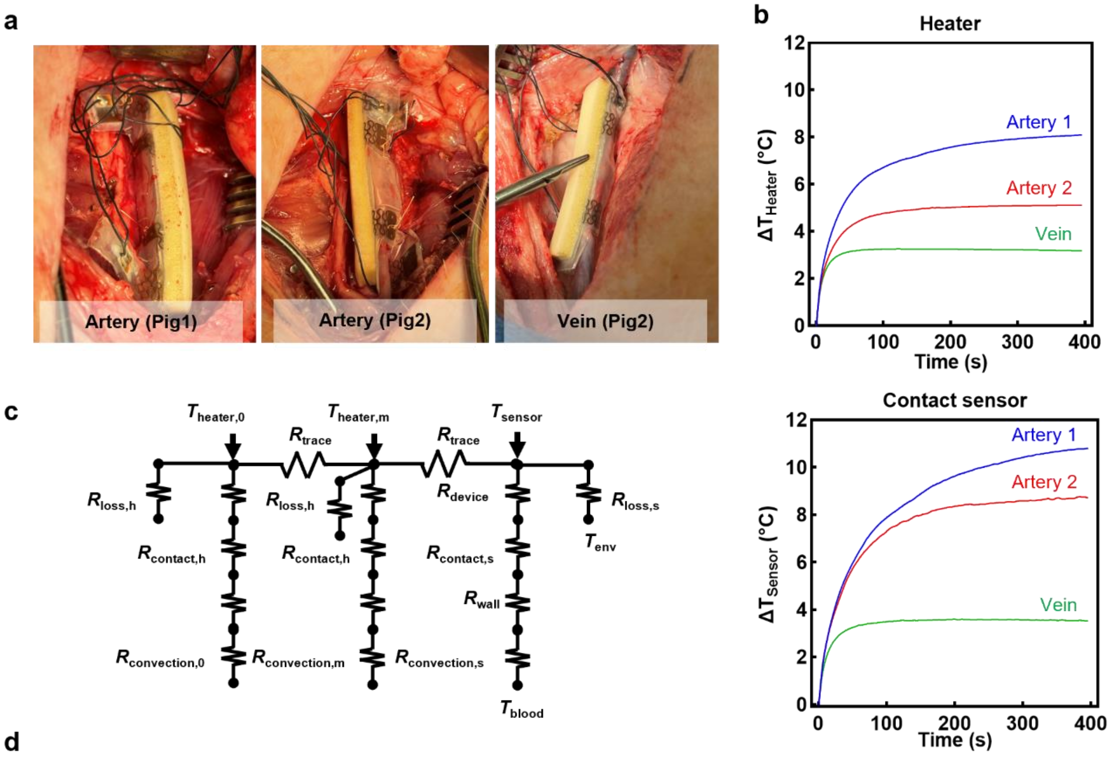

d

Thermal resistance (mm^2K/W)	Meaning	Artery 1	Artery 2	Vein
R_{trace}	Heater trace resistance		200000	
R_{device}	Device resistance		2000	
$R_{contact,h}$	Heater contact resistance	3700	1000	1000
$R_{contact,s}$	Sensor contact resistance	7500	6000	2000
R_{wall}	Vessel wall resistance	366	850	640
$R_{convection,0}$	Entry convective resistance	680	647	541
$R_{convection,m}$	Middle convective resistance	1124	1069	894
$R_{convection,s}$	End sensor convective resistance	1328	1263	1057
$R_{loss,h}$	Heater loss to environment	1e5	1e4	1e4
$R_{loss,s}$	Sensor loss to environment	1e6	1e5	1e4
$\Delta T_{heater,exp}$	Experimental heater temperature	8.1	5.1	3.2
$\Delta T_{heater,TRC}$	TRC analytical heater temperature	8.0	5.1	3.2
$\Delta T_{sensor,exp}$	Experimental sensor temperature	10.8	8.7	3.5
$\Delta T_{sensor,TRC}$	TRC analytical senor temperature	10.9	8.9	3.6

Supplementary Fig. 24: Equivalent thermal resistance circuit (TRC) analysis of in vivo experiments in a large animal model. a, Images of a device mounted on different blood vessels. The contact quality is different due to suture variability and potential arterial vasospasms. **b**, Experimental heater (top) and contact sensor (bottom) temperatures on each vessel increase as a function of time. **c**, Equivalent thermal resistance circuit (TRC) of the in vivo animal model. Heater temperature is defined as the average temperature $T_{heater} = (T_{heater,0} + T_{heater,m} + T_{heater,s})/3$. **d**, Table of thermal resistances used in the TRC analysis.

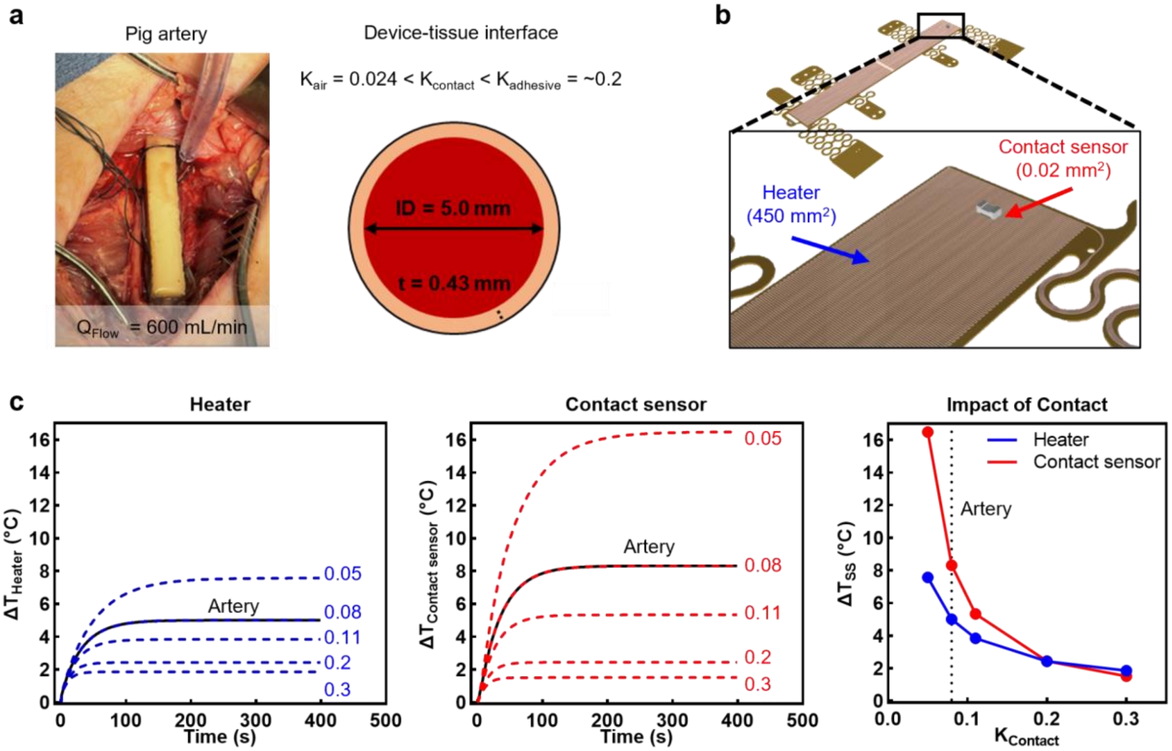

Supplementary Fig. 25: Impact of device contact with artery. a, Image of r. femoral artery of pig 2 and cross-sectional diagram of geometric parameters. Thermal conductivity at device-tissue interface ($K_{contact}$) values are estimated to be greater than that of air and less than the adhesive, which assumes perfect contact. **b**, Comparison of heater (450 mm^2) and contact sensor (0.02 mm^2) surface areas show how the larger heater better tolerates contact issues. **c**, FEA simulations (dashed lines) of heater (left) and contact sensor (middle) thermal measurements on the artery using different $K_{contact}$ values (0.05, 0.08, 0.11, 0.2, and 0.3 W/m-K). Fitted data shows good agreement with experimental measurements (black solid line) at $K_{contact}$ of 0.08. The impact of $K_{contact}$ on simulated steady state (400s) temperature rises are shown on the right.

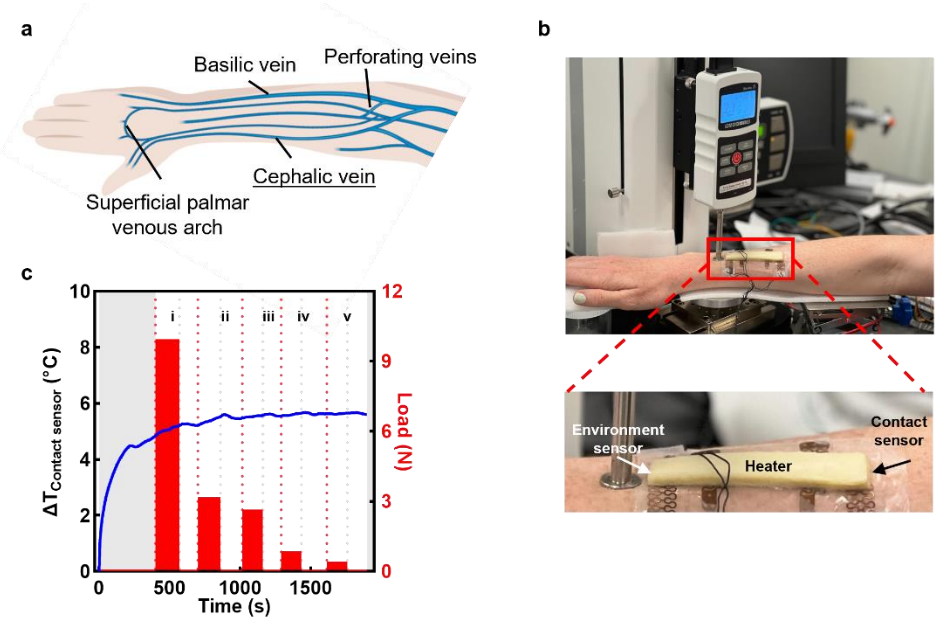

Supplementary Fig. 26: On-body demonstration of venous occlusions. a, Representative schematic of human vasculature, including the cephalic vein for testing. **b**, Image of experimental setup corresponding to main text **Fig. 4e-g**. Magnified image showing skin-mounted device in reference to POC. **c**, Contact sensor data confirming no delamination of the device. Baseline skin temperature is 32 ºC.

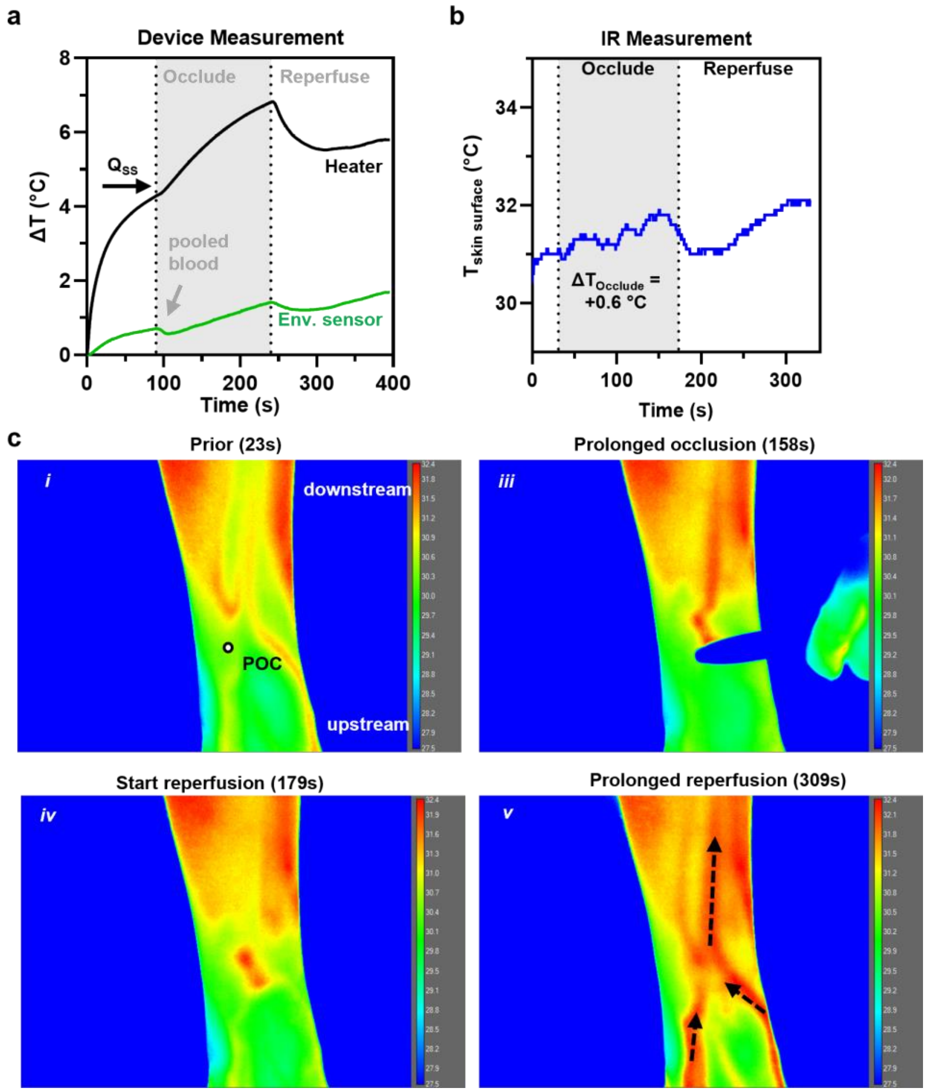

Supplementary Fig. 27: Infrared thermography (IR) of human vasculature during occlusion and reperfusion events. a, Temperature measurements of the skin surface over time, as measured by the device heater and environment sensor. Occlusion (shaded)and reperfusion (unshaded) events are labeled. **b**, IR temperature measurements of the skin surface during an identical experiment. **c**, Corresponding IR images of the forearm at (i) prior, (ii) start of occlusion, (iii) prolonged occlusion, (iv) start of reperfusion, and (v) prolonged reperfusion events. Occlusion was accomplished with compression of the cephalic vein. The direction of reperfused blood flow is indicated with black dashed arrows. Locations upstream and downstream to the point of compression (POC) are labeled. Vein compression results in the elimination of the negative pressure gradient driving venous blood flow. Blood is instead pulled into collateral vasculature deeper under the skin surface along the path of least resistance.

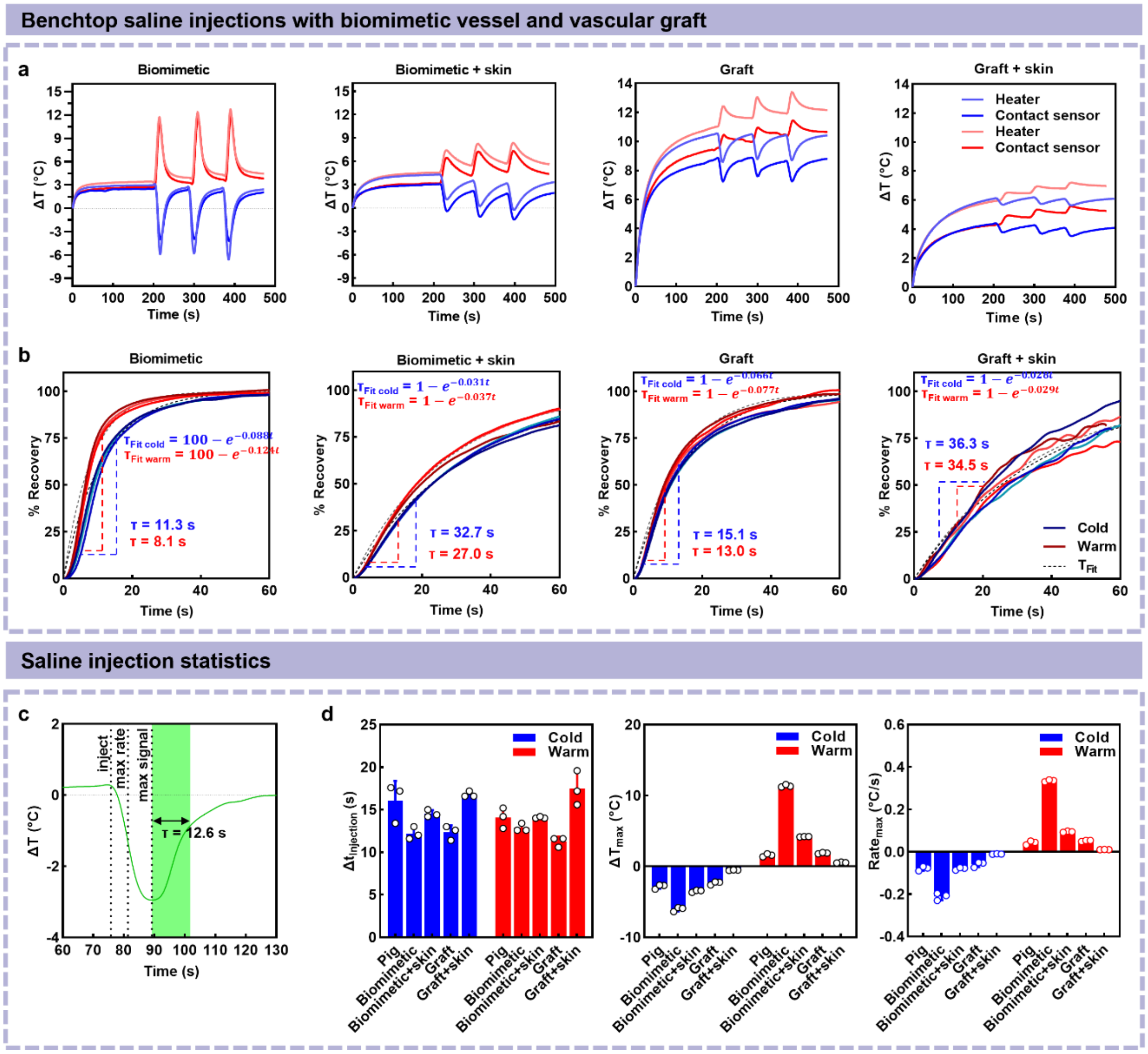

Supplementary Fig. 28: Saline injections and statistics. a, Benchtop saline injections depicting heater and contact sensor responses to cold and warm saline injections as a function of time when mounted on a biomimetic or graft vessel, with or without a layer of biomimetic skin. Environmental sensor data provided in **Fig. 5**. **b**, Percent recovery profiles for environment sensor data shown in **Fig. 5e-g**. **c**, Representative signal from environment sensor mounted on a pig artery during one cold saline injection. Labels for the point of injection, maximal rate of temperature change, and the maximal signal from injection are provided. **d**, Transient statistics for cold and warm saline injections in vivo and from benchtop models. Data is shown for (left) injection time, maximal temperature change rate, and a maximal signal from the injection. Device measurements under *patent-flow* conditions at PD 1 mW/mm^2. (n = 3, mean ± SD) .

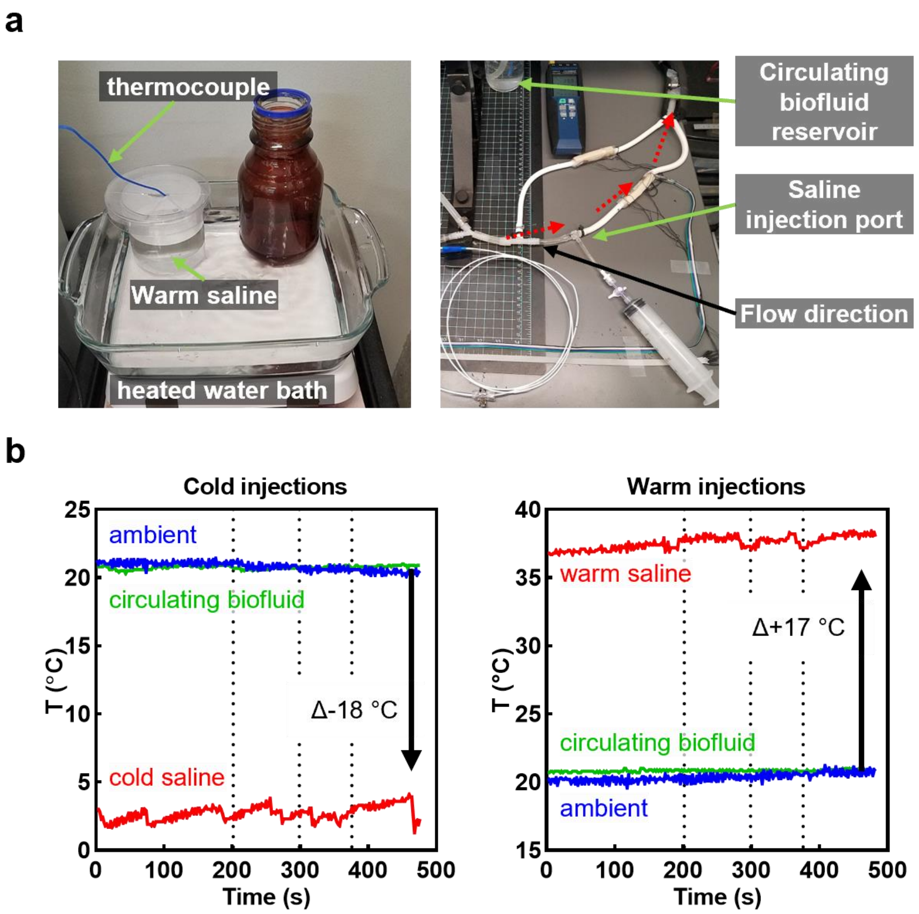

Supplementary Fig. 29: Ambient, circulating biofluid, and saline stock temperatures. a, Experimental setup for preparing temperature-controlled saline stocks and monitoring fluctuations with a thermocouple. **b**, Thermocouple measurements of ambient room temperature, circulating biofluid in the benchtop vascular models, and saline stock for both cold (left) and warm (right) saline injections. Dashed vertical lines denote the time of injections into the circulating biofluid vasculature upstream 6 cm of the device location. Note that measurements of the circulating biofluid occur in the reservoir.

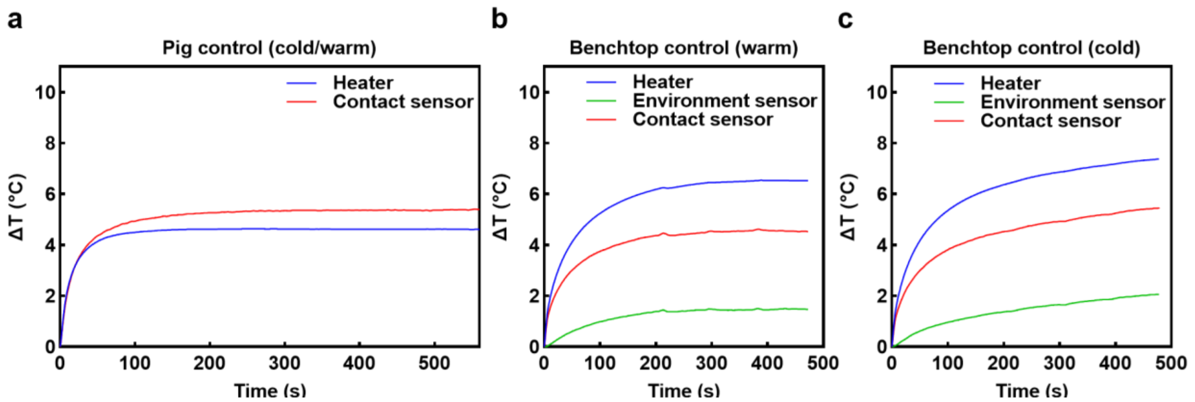

Supplementary Fig. 30: Control vessels during saline injections. **a**, *In vivo* device measurement on the pig e. jugular vein, acting as a control distal location for simultaneous saline injection and measurements on the r. femoral artery in **Fig. 5c**. **b**,**c**, Benchtop device measurements on a tributary vessel, acting as a control distal location for simultaneous saline injections and measurements on the biomimetic vessel in **Fig. 5e,f**. Data are shown for warm (**b**) and cold (**c**) saline injections. Baseline skin temperature is 32 ºC and benchtop phantom tissue temperature is 21 ºC.

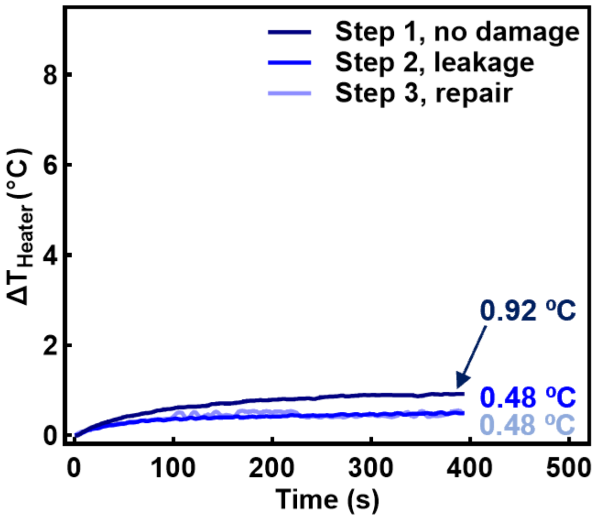

Supplementary Fig. 31: Contact sensor during venous leakage and repair. Corresponds with **Fig. 5h**.

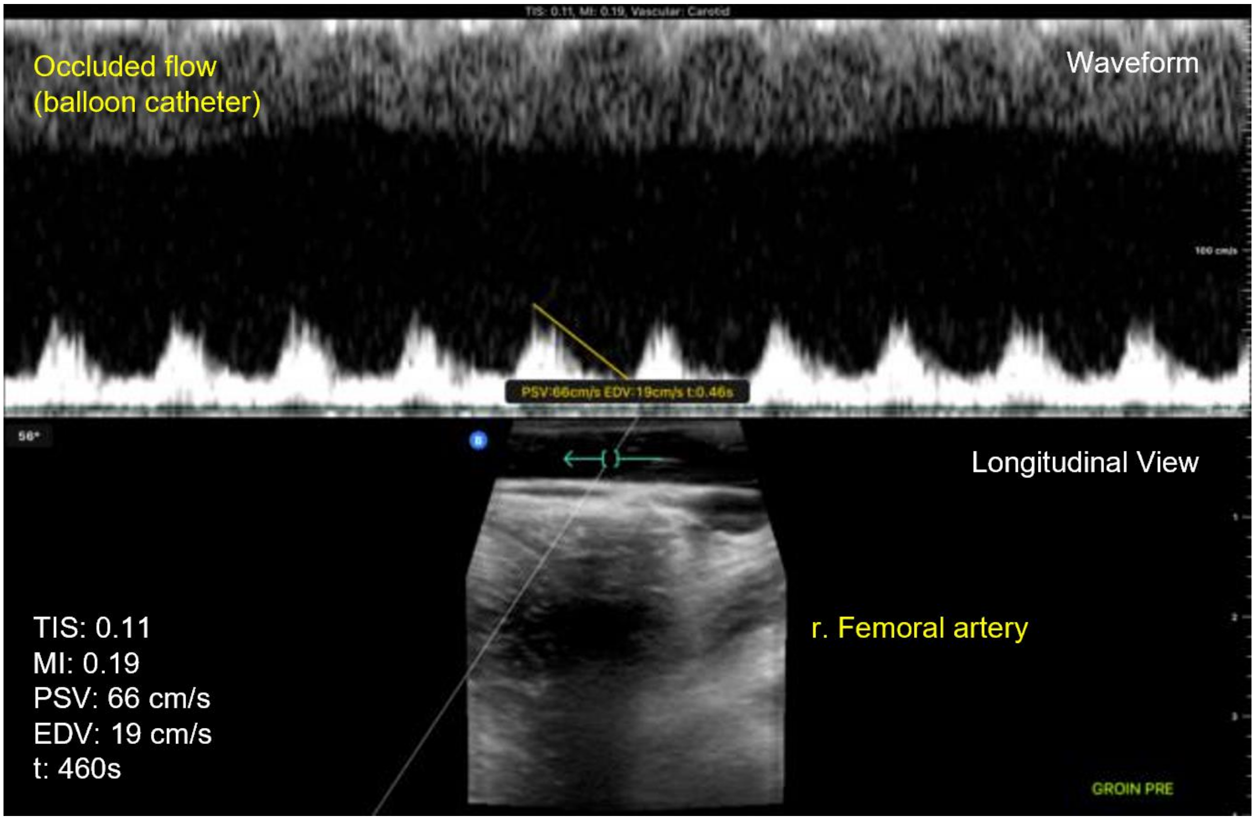

Supplementary Fig. 32: Duplex ultrasound during balloon catheter occlusion. US waveform (top) and longitudinal view (bottom) of the r. femoral artery at a distal location during a balloon catheter occlusion. Labels for TIS (thermal index for soft tissue), MI (mechanical index), PSV (peak systolic velocity), EDV (end-diastolic velocity), and t (pulse time).

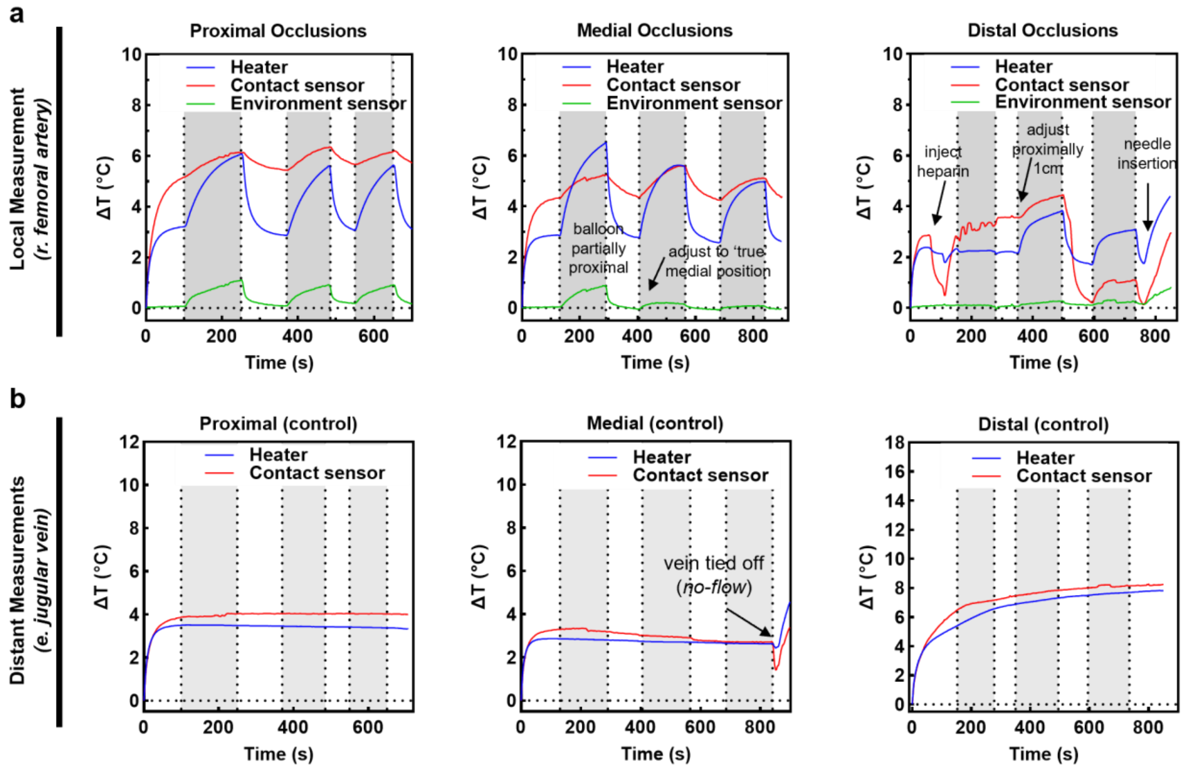

Supplementary Fig. 33: Raw data for balloon catheter occlusions of the r. femoral artery. a, *In vivo* data from the heater, contact sensor, and environment sensor in a device mounted on the r. femoral artery. **b**, Simultaneous measurements with a second device mounted on the e. jugular vein to act as a control vessel. Environmental sensor data not shown due to electrical disconnection. Occlusions performed on the r. femoral artery at positions proximal, medial, and distal to the local measurement device (n=1, occlusions in triplicate). Dashed vertical lines denote time of occlusion and reperfusion. Corresponds with **Fig. 6d**. Baseline vessel surface temperatures range from 34-37 ºC.

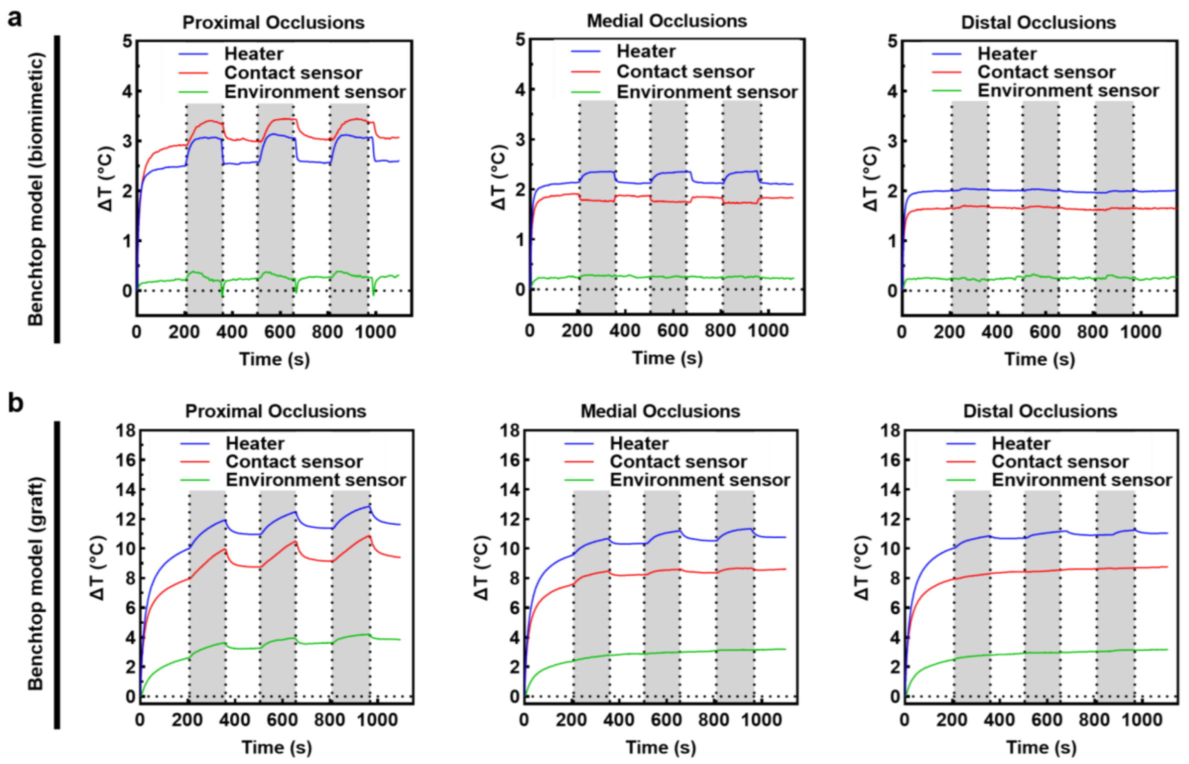

Supplementary Fig. 34: Raw data for balloon catheter occlusions on benchtop models. a, Benchtop data from the heater, contact sensor, and environment sensor in a device mounted on the biomimetic vessel. **b**, Benchtop data from the heater, contact sensor, and environment sensor in a device mounted on the graft vessel. Occlusions were performed on each vessel at proximal, medial, and distal positions to the device (n=3, occlusions in triplicate). Dashed vertical lines denote times of occlusion and reperfusion. Note that longer equilibration times (200 s) are used for benchtop models in contrast to *in vivo* results to account for the lower k higher t synthetic graft. Data correspond to **Fig. 6f-g**. Baseline benchtop phantom tissue temperature is 21 ºC.

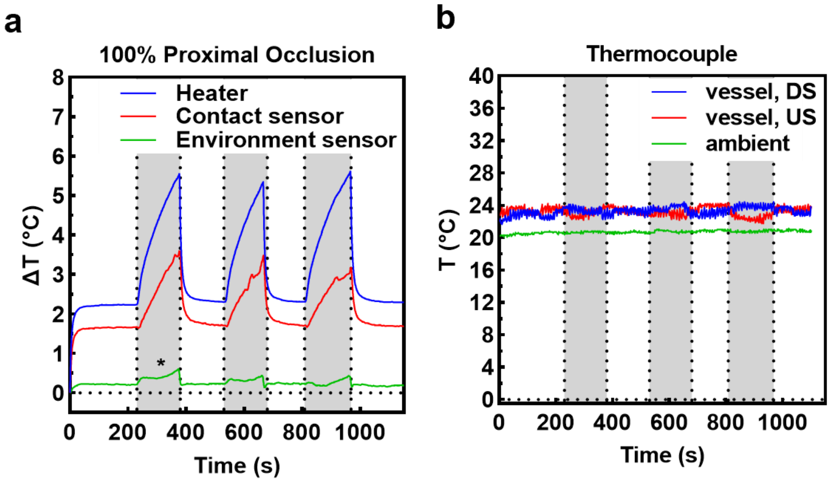

Supplementary Fig. 35: Raw data for balloon catheter 100% occlusion of a biomimetic vessel. a, Real-time flow sensing during successive occlusion (no-flow) and reperfusion (patent-flow) events with the biomimetic vessel at proximal location (n=1 in triplicate). This contrasts with 90% occlusions in **Fig. 6f**. **b**, Thermocouple measurements of ambient room temperature, and biomimetic vessel surface temperature at downstream (DS) and upstream (US) positions of the device. Dashed vertical lines denote times of occlusion and reperfusion. Baseline benchtop phantom tissue temperature is 21 ºC.

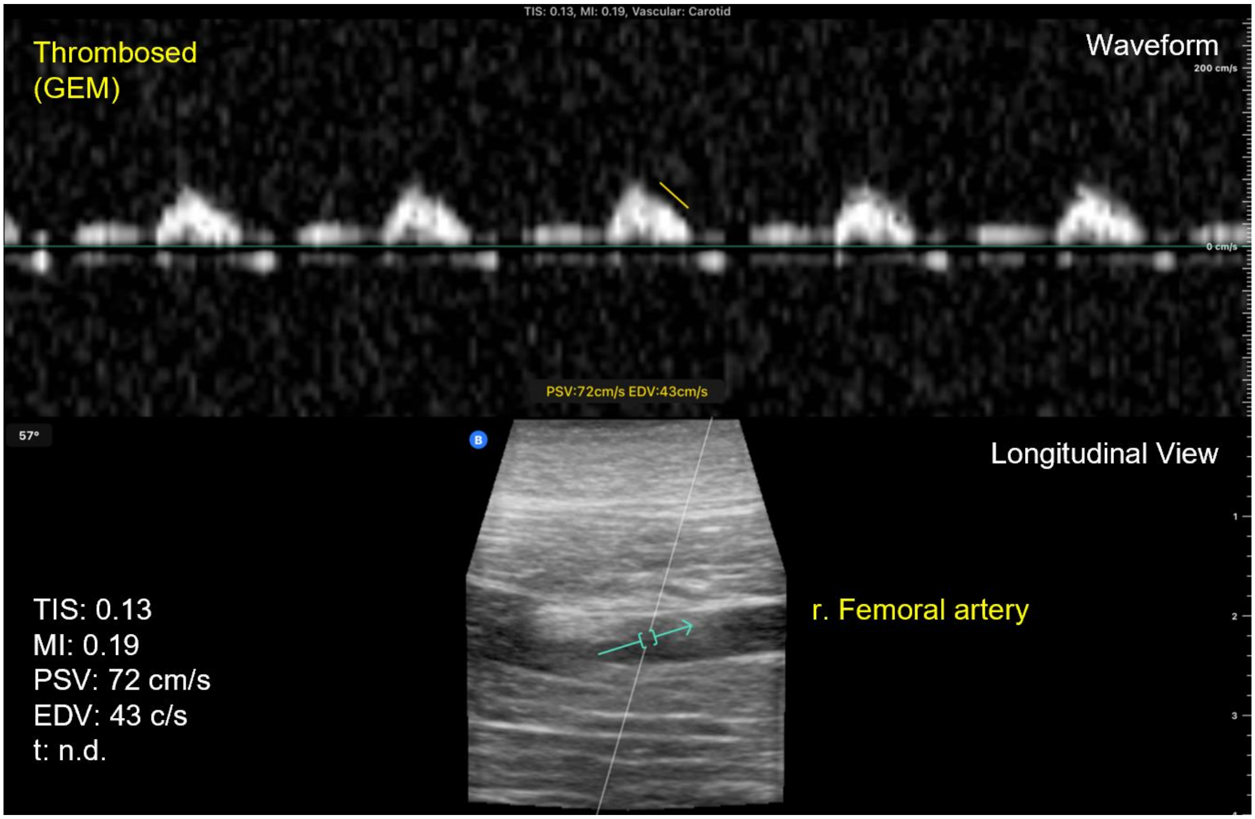

Supplementary Fig. 36: Duplex ultrasound during thrombosis. US waveform (top) and longitudinal view (bottom) of the r. femoral artery at a distal location during a thrombosis event. Labels for TIS (thermal index for soft tissue), MI (mechanical index), PSV (peak systolic velocity), EDV (end-diastolic velocity), and t (pulse time).

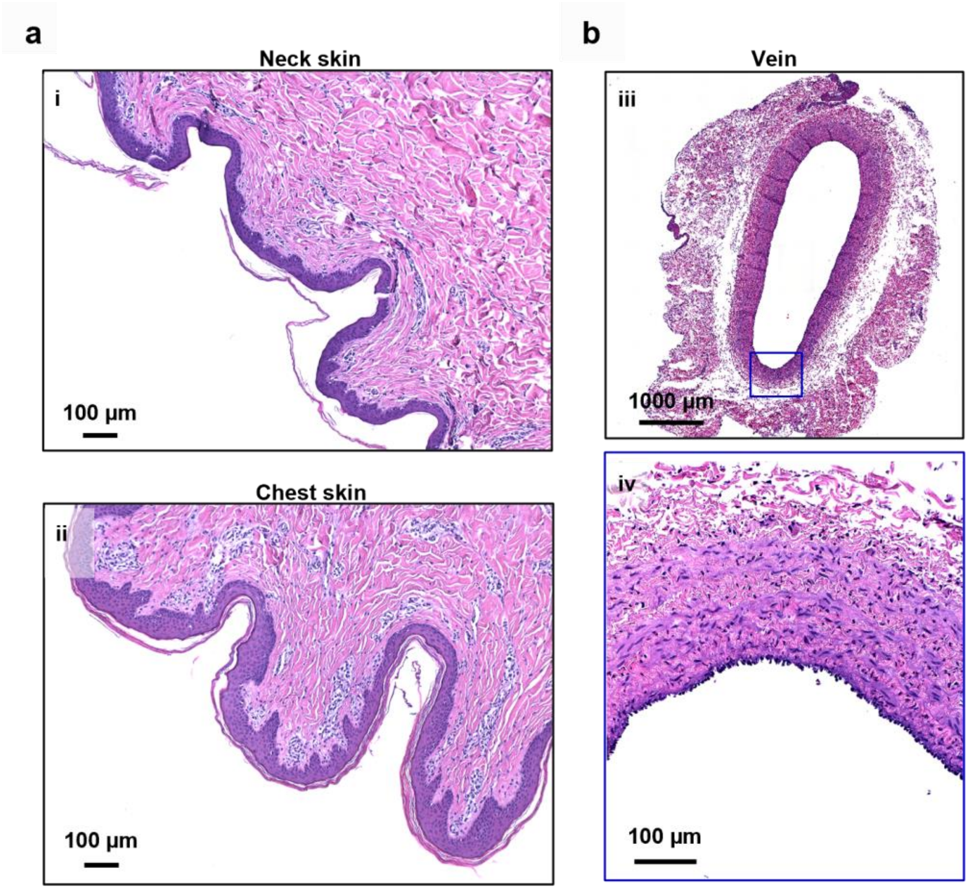

Supplementary Fig. 37: Histology of in vivo tissue. Hematoxylin and eosin (H&E) stains of excised porcine tissue after mounted device testing. **a**, Histology of neck (i) and chest (ii) skin. **b**, Histology of the e. jugular vein (iii) and corresponding magnified view (iv) below outlined in blue. Scale 100 or 1000 µm.

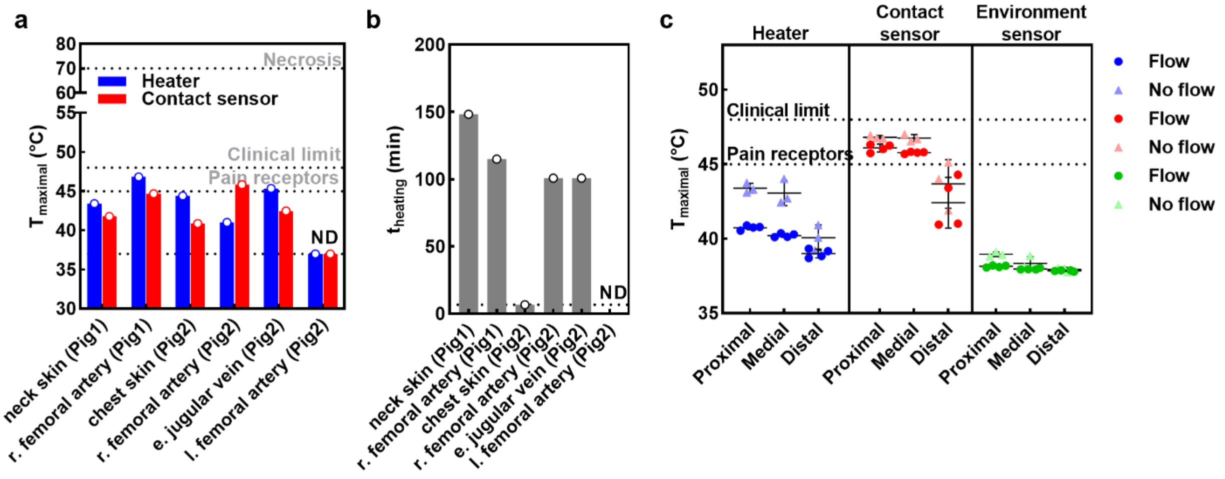

Supplementary Fig. 38: In vivo biocompatibility of thermal dosing. a, Maximal temperature rise from the heater and contact sensor during measurements at various test locations. Labels denote temperatures at which tissue necrosis, the clinical limit for transdermal devices, and the initial activation temperature for pain receptors occur. **b**, Total time of heating measurements that each tissue was exposed to in this study. Corresponds to tissues analyzed by histology in **Fig. 7**. **c**, Maximal temperature rise from the heater, contact sensor, and environment sensor during balloon catheter occlusions in **Fig. 6**. (n = 3-4, mean ± SD) Baseline tissue temperatures are reported in **Supplementary Fig. 23**.

References

1 Oglat, A. A. *et al.* A Review of Medical Doppler Ultrasonography of Blood Flow in General and Especially in Common Carotid Artery. *J Med Ultrasound* **26**, 3-13 (2018). https://doi.org:10.4103/jmu.Jmu_11_17

2 Wang, F. *et al.* Flexible Doppler ultrasound device for the monitoring of blood flow velocity. *Science Advances* **7**, eabi9283 (2021). https://doi.org:doi:10.1126/sciadv.abi9283

3 Monnet, X. & Teboul, J.-L. Transpulmonary thermodilution: advantages and limits. *Critical Care* **21**, 147 (2017). https://doi.org:10.1186/s13054-017-1739-5

4 Franklin, D. *et al.* Synchronized wearables for the detection of haemodynamic states via electrocardiography and multispectral photoplethysmography. *Nature Biomedical Engineering* **7**, 1229-1241 (2023). https://doi.org:10.1038/s41551-023-01098-y

5 Boutry, C. M. *et al.* Biodegradable and flexible arterial-pulse sensor for the wireless monitoring of blood flow. *Nature Biomedical Engineering* **3**, 47-57 (2019). https://doi.org:10.1038/s41551-018-0336-5

6 Mun, C. H. *et al.* Three-Dimensional Electrospun Poly(Lactide-Co-ε-Caprolactone) for Small-Diameter Vascular Grafts. *Tissue Engineering Part A* **18**, 1608-1616 (2012). https://doi.org:10.1089/ten.tea.2011.0695

7 Lauvao, L. S. *et al.* Vein diameter is the major predictor of fistula maturation. *Journal of Vascular Surgery* **49**, 1499-1504 (2009). https://doi.org:https://doi.org/10.1016/j.jvs.2009.02.018

8 Hasgall, P. A. *et al.* Vol. 4.1 (IT'IS Foundation, 2022).

9 Foor, J. S., Moureau, N. L., Gibbons, D. & Gibson, S. M. Investigative study of hemodilution ratio: 4Vs for vein diameter, valve, velocity, and volumetric blood flow as factors for optimal forearm vein selection for intravenous infusion. *The Journal of Vascular Access* **25**, 140-148 (2024). https://doi.org:10.1177/11297298221095287

10 Mukai, K. *et al.* Safety of Venipuncture Sites at the Cubital Fossa as Assessed by Ultrasonography. *J Patient Saf* **16**, 98-105 (2020). https://doi.org:10.1097/pts.0000000000000441

11 MacRae, J. M. *et al.* Arteriovenous Vascular Access Selection and Evaluation. *Can J Kidney Health Dis* **3**, 2054358116669125 (2016). https://doi.org:10.1177/2054358116669125

12 Edwards, J. *et al.* The functional vascular anatomy of the swine for research. *Vascular* **30**, 392-402 (2022). https://doi.org:10.1177/1708538121996500

13 Krishnamoorthy, M. K. *et al.* Anatomic configuration affects the flow rate and diameter of porcine arteriovenous fistulae. *Kidney International* **81**, 745-750 (2012). https://doi.org:https://doi.org/10.1038/ki.2011.468

14 Liang, X. M. *et al.* High accuracy thermal conductivity measurement of aqueous cryoprotective agents and semi-rigid biological tissues using a microfabricated thermal sensor. *Scientific Reports* **5**, 10377 (2015). https://doi.org:10.1038/srep10377
