# Supplementary material for: A soft thermal sensor for the continuous assessment of flow in vascular access
Source: Nat Commun. 2025 Jan 2;16:38. doi: 10.1038/s41467-024-54942-3 (PMC11696732; doi:10.1038/s41467-024-54942-3)
Supplement: Supplementary file 2 — Description of Additional Supplementary Files [file 41467_2024_54942_MOESM2_ESM.docx]

**Description of Additional Supplementary Files**

Supplementary movie 1: IR Arm Occlusions.

Supplementary movie 2: Patent Flow (unobstructed)

Supplementary movie 3: Thrombosis (obstructed)

Supplementary Code: Custom LabVIEW Software and Data
